# Effect of Hydrophobic Cross-Linkers in Strong Base Gel-Type Resins on the Adsorption Kinetics and Capacity for Perfluoroalkyl Substances

**DOI:** 10.1021/acsestwater.5c00094

**Published:** 2025-06-13

**Authors:** Florian Junge, Fiona E. Rückbeil, Regina Gnirss, Rainer Haag, Alejandro Lorente, Fabio Lorenz, Sunil P. M. Menacherry, Aki S. Ruhl, Alexander Sperlich, Ana Zidar, Olaf Wagner

**Affiliations:** † 506296 Berliner Wasserbetriebe, Neue Jüdenstraße 1, 10179 Berlin, Germany; ‡ Institut für Chemie und Biochemie, 9166 Freie Universität Berlin, Arnimallee 22, 14195 Berlin, Germany; § 26524 Technische Universität Berlin, Water Treatment, KF4, Fasanenstraße 1A, 10623 Berlin, Germany; ∥ German Environment Agency, Section II 3.3, Schichauweg 58, 12307 Berlin, Germany

**Keywords:** PFAS adsorption, water treatment, ion exchange resins, fluorine−fluorine interactions, siloxane, polyethylenimine

## Abstract

The persistence and water mobility of per- and polyfluoroalkyl substances (PFAS) have led authorities worldwide to lower regulatory limits to prevent adverse health effects. Removal via adsorption on activated carbon can be inefficient due to the unspecific surface interaction, while ion exchange resins with positive charges and hydrophobic chains can offer faster kinetics and improved removal. In here, novel cationic resins were synthesized by cross-linking polyethylenimine, followed by methylation. To obtain cross-linked particles and introduce hydrophobic interacting moieties in one single synthetic step, aliphatic, fluorous, and silicone-based oligoethers were used as cross-linkers. These cationic adsorbents were compared with two state-of-the-art strong base gel-type ion exchange resins and granular activated carbon in isotherm and kinetic studies. The newly developed adsorbents showed significantly faster removals of all tested long- and short-chain PFAS. The fluorous cationic adsorbent achieved equilibrium loadings that were comparable to those of the state-of-the-art adsorbents for all PFAS with five or more perfluorinated carbon atoms.

## Graphical abstract

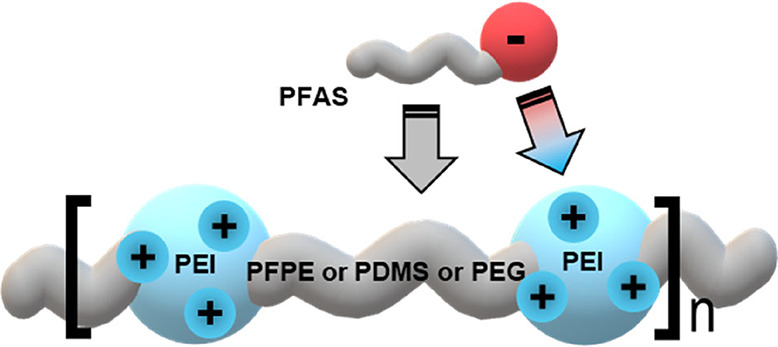

## Introduction

The excessive, unrestricted use of fluorinated organic molecules and fluoropolymers resulted in a global contamination of water, soil, and organisms with per- and polyfluoroalkyl substances (PFAS). The high persistence, negative health effects, and mobility in the water cycle of many PFAS have led to several countries, for example, in North America and Europe, introducing strict limits on the ng/L level in drinking, surface, and groundwater.^1,2^ These new limit values represent a major challenge for water treatment. The most commonly used process at present is adsorption on granular activated carbon (GAC) in fixed-bed filters.^3,4^ In complex real waters, adsorptive removal by GAC can be uneconomical, as competing adsorption of other water constituents prevents selective removal, and only low capacities and early breakthroughs are achieved, especially for short-chain PFAS.^5,6^ In addition, long retention times with correspondingly large systems are required due to slow kinetics. Faster kinetics and improved removal of short-chain PFAS can be achieved through the use of ion exchange materials (IX), which are increasingly being used successfully for PFAS removal on an industrial scale.^7^ The hydrophobic alkyl chains or the polystyrene backbone of the IX itself support the binding of the hydrophobic fluoroalkyl tail groups, while the positive charge of these resins binds the head groups of the predominantly negatively charged PFAS, most notably perfluorocarboxylic acids (PFCA) and perfluorosulfonic acids (PFSA).^8^

The most highly positively charged polymers are the protonated or methylated derivatives of branched polyethylene imine (bPEI), linear polyethylene imine (lPEI), and poly­(vinylamine) (PVAm) because they possess the highest content of amine groups of any polymer. The synthesis of PVAm and lPEI requires the use of protecting groups to prevent imine tautomerization or branching, respectively.^9,10^ Thus, bPEI remains the only one of the three polymers that is accessible in large scale through a one-step cationic polymerization. Hyperbranched structures, like those found in bPEI, are also known for their generally superior multivalent activity compared to linear polymers,^11^ which made them promising candidates to be tested as PFAS adsorbents.^12–16^ However, pristine bPEI is unsuitable as an adsorbent as it is a water-soluble liquid.^10^ Bi- or oligofunctional electrophiles are commonly used to cross-link bPEI to obtain insoluble cross-linked PEI (cPEI) particles.^17–23^ Besides the high density of potential cationic ammonium groups in cPEI, the benefit of using cPEI as an adsorbent is the possibility to efficiently introduce different hydrophobic moieties during the cross-linking step. Potential cross-linkers include di- or polyaldehydes,^18,22,24–29^ epichlorohydrin,^23^ diisocyanates,^21,30–33^ dihalides,^17,19,24,34^ diacrylates,^35^ di-*N*-hydroxysuccinimide esters^36^ and di- or triepoxides.^12,20,22,35,37–39^ However, it is beneficial to retain a high amount of amine groups during the cross-linking of bPEI since only amines can be protonated or alkylated to positively charged ammonium groups subsequently. Diepoxides are therefore the only type of potential cross-linkers that preserve amines as functional groups without requiring the addition of any base or reducing agents and without the formation of hydrolysis-prone linkers. Further, many diepoxides, especially diglycidyl ethers, are commercially available or can be conveniently prepared from commercial precursors. In contrast to the industrially used butylated ammonium groups,^40^ we chose to create the quaternary ammonium groups on the PEI-based materials through methylation due to steric repulsion. Noteworthy, two recent studies observed that either trimethylated fluorinated^41^ or dodecyldimethylammonium-based adsorbents^42^ tend to have better PFAS adsorption than their tributylated derivatives.

Highly fluorinated alkyl chains interact with each other in the form of specific fluorine-fluorine interactions.^43^ Hence, modification of adsorbents with fluoroalkyl groups, preferably in the polymer backbone than in the side chain,^44^ is a common and largely successful design principle to boost the adsorption of PFAS.^45,46^ Nevertheless, the effectiveness of fluorophilic modification of PFAS adsorbents is still subject of scientific debate^47,48^ and PFAS removal technologies should ideally avoid introducing additional PFAS-functionalized materials into the environment. Thus, we developed not only a fluorous low-molecular-weight perfluoropolyether (PFPE)-cPEI but also two more sustainable, fluorine-free cPEI adsorbents with aliphatic poly­(ethylene glycol) (PEG) and silicone-based poly­(dimethylsiloxane) (PDMS) cross-linkers for comparison. This is, to the best of our knowledge, the first IX with dimethylsiloxane functional groups applied for PFAS removal. There have been few examples of PEI/poly­(dialkyl siloxane) composites being used for other applications.^49–52^ PDMS shares some properties with fluoropolymers, such as low surface energies, water-repellency, and good thermal stability.^53,54^ Herein, we wanted to investigate whether PDMS provides a suitable hydrophobic group for the selective removal of PFAS. Molecular dynamic calculations from Ke et al.^55^ suggested hydrophobic-driven adsorption of PFSA onto inorganic siloxane surfaces (kaolinite), indicating a potential viability of this concept.

As the industry started replacing long-chain PFAS with shorter ones, a growing contamination with short-chain PFAS,^56–59^ which are by definition of the Organisation for Economic Co-operation and Development (OECD)^60^ all PFCA and PFSA with less than six fluorinated carbon atoms arises. Consequently, the efficient removal of short-chain PFAS becomes a challenge of increasing importance. Therefore, we chose to focus on four short-chain PFAS (trifluoromethanesulfonic acid, TFMSA; perfluoropentanesulfonic acid, PFPeS; perfluorobutanoic acid, PFBA; and perfluorohexanoic acid, PFHxA) as analytes. We investigated the adsorption isotherms and the kinetics of their adsorption onto PFPE, PDMS, and PEG cross-linked PEI and benchmarked them against the performance of a commercialized, state-of-the-art IX for PFAS removal.

## Methods

### Materials

Sodium hydride (60 wt % dispersion in mineral oil), 15-crown-5 (98%), bis­(3-(oxiran-2-ylmethoxy)­propyl)-terminated polydimethylsiloxane (PDMS cross-linker, product number: 480282, *M*_n_ = 800 g/mol), sodium bicarbonate (NaHCO_3_, 99.5%), sodium chloride for the model solution (NaCl, 99%), and polyethylene glycol diglycidyl ether (PEGDE, product number: 475696, *M*_n_ = 500 g/mol) were purchased from Sigma-Aldrich (Taufkirchen, Germany). Branched polyethylenimine (bPEI, catalogue number: 19850, *M*_w_ = 10 000 g/mol) was purchased from PolySciences (Hirschberg an der Bergstrasse, Germany). Dimethyl sulfate (99%), 18-crown-6 (99%), *m*-CPBA (70% - 75% in water), potassium iodide (99%), dry THF (99.5%), sodium chloride for the ion-exchange (99.5%), aqueous ammonia solution (25 w%), THF (99.6%), methanol (>99%), and allyl bromide (98%) were purchased from Acros Organics/Thermo Fisher Scientific (Schwerte, Germany). 2,2’-((Oxybis­(1,1,2,2-tetrafluoroethane-2,1-diyl))­bis­(oxy))­bis­(2,2-difluoroethan-1-ol) (fluorinated tetraethylene glycol, 98%) was purchased from abcr (Karlsruhe, Germany). Potassium carbonate (K_2_CO_3_, 99%) and sodium sulfate (Na_2_SO_4_, 99%) were purchased from Carl Roth (Karlsruhe, Germany). DCM (≈100%) and propan-2-ol (Reag. Ph. Eur.) were purchased from VWR (Darmstadt, Germany). Argon (Alphagaz, 99.999%) was purchased from Air Liquide (Düsseldorf, Germany). Deuterated chloroform (99.8%) was purchased from Deutero (Kastellaun, Germany). Sodium trifluoromethanesulfonate (95%) was purchased from Toronto Research Chemicals (Toronto, Canada). All other PFAS standards were purchased from Campro Scientific (Berlin, Germany): PFBA (99.8%), PFHxA (99%), perfluoroheptanoic acid (PFHpA, 96%), perfluorooctanoic acid (PFOA, 95%), perfluorononanoic acid (PFNA, 95%), perfluorodecanoic acid (PFDA, 99.9%), perfluorobutanesulfonic acid (PFBS, 97%), PFPeS (98%), sodium perfluorohexanesulfonate (PFHxS, 98%), and potassium perfluorooctanesulfonate (PFOS, 99.8%). All compounds were used as received.

### Commercialized Adsorbents

The bituminous GAC Hydraffin 30N^61^ was purchased from Donau Carbon (Frankfurt, Germany). The two polystyrene strong base anion exchange resins PFA694E^62^ and Lewatit TP108 ^63^ were purchased from Purolite (Philadelphia, United States) and Lanxess (Cologne, Germany).

### Synthetic Procedures

#### Fluorinated Tetraethylene Glycol Diglycidyl Ether (PFPE Cross-Linker)

Sodium hydride (NaH, 60 wt % in mineral oil, 2.93 g, 3.0 equiv) was added to a Schlenk flask with dry tetrahydrofuran (THF, 80 mL). Fluorinated tetraethylene glycol (9.99 g, 1.0 equiv) was added slowly (intense foam development!) to the NaH/THF mixture under ice cooling. After the addition of allyl bromide (6.32 mL, 3.0 equiv), the mixture was heated to reflux for 24 h. The reaction was terminated by the addition of propan-2-ol (6 mL) and water (5 mL) at 0 °C. The organic solvent was evaporated, and the residue was diluted with water (50 mL) and dichloromethane (DCM, 100 mL). The phases were mixed thoroughly. The organic layer was separated, washed with water (2 × 50 mL), and *m*-CPBA (purity: 70–75%, with remaining water and *meta*-chlorobenzoic acid, 23.2 g, 4.0 equiv) was added. The reaction mixture was stirred for 4 days at room temperature. The reaction mixture was washed with saturated aqueous sodium bicarbonate solution (4 × 100 mL) and water (2 × 100 mL), followed by drying with Na_2_SO_4_, filtration, and evaporation of solvent. The crude product was purified by column chromatography (silica, hexane/ethyl acetate 2/1 → 0/1). The PFPE cross-linker (9.83 g, 77%) was obtained as a slightly yellow liquid. ^1^H NMR (400 MHz, CDCl_3_): δ = 3.97–3.85 (m, 6H), 3.52 (dd, *J* = 11.5 Hz, 6.0 Hz, 2H), 3.16–3.13 (m, 2H), 2.79 (t, *J* = 4.5 Hz, 2H), 2.61 (dd, *J* = 5.0 Hz, 2.6 Hz, 2H) ppm. ^13^C NMR (101 MHz, CDCl_3_): δ = 122.4 (t), 116.9–112.3 (m), 73.0, 70.0, 49.5, 39.8 ppm. ^19^F NMR (376 MHz, CDCl_3_): δ = −77.9 (4F), −88.6 (4F), −88.9 (4F) ppm. ESI-ToF-MS *m*/*z* = 545.0416, C_14_H_14_F_12_O_7_Na_1_^+^: calc. 545.0446. elemental analysis (%) C 32.59 H 2.73 N 0.14 S 0.00, calc. C 32.20, H 2.70, N 0, S 0.

#### General Procedure of the Cross-Linking of PEI

bPEI was dissolved in THF (70 mL), and an equal mass of the respective cross-linker was added. The solution was refluxed for 24 h (PFPE and PEG cross-linker), while the reaction time was extended to 42 h in the case of the PDMS cross-linker because no solidification occurred after 24 h. The cross-linked polymer was separated and washed with methanol (2 × 80 mL; PDMS-cross-linked polymer: 2 × 40 mL) in a centrifuge. The unquaternized PEG-cross-linked polymer was further washed with DCM (2 × 80 mL). The unquaternized PFPE- and PDMS-cross-linked polymers could not be successfully separated from the DCM via centrifugation or filtration. The cross-linked polymers were then dried and ground in liquid nitrogen.

#### Unquaternized PEG-Cross-Linked PEI (uPEG-cPEI)

The reaction was performed according to the general procedure described above with bPEI (11.1 g) and PEGDE (9.69 mL). Unquaternized PEG-cross-linked PEI (uPEG-cPEI) (21.1 g) was obtained as an elastic colorless solid.

#### Unquaternized PDMS-Cross-Linked PEI (uPDMS-cPEI)

The reaction was performed according to the general procedure described above with bPEI (10.9 g) and the commercial PDMS cross-linker (11.0 mL). Unquaternized PDMS-cross-linked PEI (uPDMS-cPEI) (20.7 g) was obtained as an elastic colorless solid.

#### Unquaternized PFPE-Cross-Linked PEI (uPFPE-cPEI)

The reaction was performed according to the general procedure above with bPEI (8.99 g) and PFPE cross-linker (8.99 g). Unquaternized PFPE-cross-linked PEI (uPFPE-cPEI) (20.7 g) was obtained as an elastic colorless solid.

#### Methylation of Unquaternized, Cross-Linked PEIs

The respective unquaternized, cPEI (10.0 g), and K_2_CO_3_ (8.00 g, 57.9 mmol, 0.5 equiv regarding the ethylenimine repeating unit under assumption of 50 wt % PEI content) were added to dimethyl sulfate (100 mL, 1.05 mol, 9.1 equiv) in a flame-dried Schlenk flask. The reaction was stirred for 3 days at room temperature before it was carefully stopped (retarded runaway reaction possible!) by the portion wise addition of 25% aqueous ammonia solution (100 mL) at 0 °C. The polymer was filtered off and washed with water (100 mL). Alternatively, centrifugation might be used for separation, but decantation of water is challenging for the nonfluorinated polymers. The polymers were dried and suspended together with K_2_CO_3_ (8.00 g) under argon in dimethyl sulfate (100 mL). The reaction mixture was allowed to stir for another 4 days at room temperature. The reaction was quenched and washed with water, as described previously. Additionally, the polymers were washed with THF (2 × 50 mL) and then stirred in saturated brine (450 mL) for 1 day at room temperature. The polymers were filtered off, washed with water (3 × 50 mL), and dried at 100 °C. Grinding in liquid nitrogen gave PFPE-cPEI (12.9 g) as yellowish powder, PDMS-cPEI (9.05 g) as white powder, and PEG-cPEI (12.2 g) as brown coarse particles. All samples contained a few elastic transparent chunks that could not be successfully grinded. The particles were sieved directly after drying in high vacuum as dry powder (the particles adsorb moisture within minutes) with two analysis sieves (mesh sizes: 710 and 63 μm; particles outside of this range were cutoff) from Retsch (Haan, Germany) before optical microscopy, PFAS adsorption, BET and SAXS analysis.

#### Optical Microscopy

Optical microscopy images were taken in Milli-Q water with an Axio Observer.Z1 from Carl Zeiss (Jena, Germany) equipped with an objective EC Plan-Neofluar 5x/0.16 M27 (magnification 5 x) or LD Achroplan 20x/0.40 Korr Ph 2 (magnification 20 x) and an AxioCamMRm3 camera with a 0.63x adapter. Images of 1388 × 1040 pixels were recorded. The software for capturing the images was the ZEN 2012 (blue edition) version 1.1.2.0 from Carl Zeiss, and for particle size analysis, the Fiji^64^ version of ImageJ 1.54k was used. The particle contours of each particle on one (PFPE-cPEI and PEG-cPEI), three (PDMS-cPEI), or five images (TP108) (Figures S18–S21) were measured manually with the polygon tool due to the heterogeneous background and difficulties with the automatic detection of the particles. Particles that were vastly out of the focus plane, too small to be identified clearly, partially outside the pictured area, or overlapped extensively with other particles were not analyzed. In some instances, it was hard to differentiate between an agglomerate and a single particle due to the complex particle shapes. These were counted as one particle. Either the Feret diameter (Figures S22a–S25a), the minFeret diameter (Figures S22b–S25b) or the projected area diameter *d* calculated from the area of the drawn polygons *A* reductively assuming spherical particles [*d* = 2 * √(*A*/π)] (Figures 2b–d and S22c) were reported in number-weighted distribution plots.

### Adsorption Experiments

#### Model Solution Preparation and Sample Processing

All adsorption experiments were carried out in simple model solutions. For this purpose, ultrapure water (Milli-Q) was mixed with the buffer and electrolytes (0.05 mM NaHCO_3_ and 0.02 mM NaCl). PFAS were added to the model solution from high concentrated stock solutions (10 mg/L) to achieve the desired starting concentration of 10 μg/L. The pH of the model solution was adjusted to a neutral pH value (7 ± 0.2) by adding 0.1 M NaOH and HCl. All adsorption experiments were conducted at room temperature (approximately 22 °C).

After the respective contact time and prior to analysis, all samples were filtered using prerinsed 0.45 μm membrane filters made of regenerated cellulose acetate (Macherey-Nagel, Düren, Germany). Samples were stored at 4 °C in 5 mL polypropylene vials purchased from Th. Geyer (Renningen, Germany).

#### Screening Experiment

The three cPEI adsorbents were compared with Hydraffin 30N GAC and PFA694E IX in an initial screening experiment. For this purpose, 40 mg of adsorbent (dry weight) was added to 1 L of the model solution and contaminated with a PFAS mix containing PFHpA, PFOA, PFNA, PFDA, PFBS, PFHxS, and PFOS. Samples were agitated in 1 L glass bottles on a horizontal shaker, which were sealed with polypropylene screw caps. All batches were prepared in duplicate, and the first duplicate was agitated for 30 min (pre-equilibrium) and the second for 10 days (equilibrium).

The percentage removal was determined by eq 1 after the respective contact time *t* by comparing the remaining PFAS concentration in the model solution (*c*) with the concentration in a reference sample without adsorbent (*c*_0_), which was otherwise treated in the same way.

$$\mathrm{removal}=100\%\cdot \left(1-\frac{c(t)}{c_{0}(t)}\right)$$ 1

Single point adsorption coefficients *K*_d_ were calculated using eq 2 to allow better comparability within the data sets of this study and other studies.

$$K_{d}=\frac{q(t)}{c(t)}$$ 2

Hereby, parameter *q* describes the PFAS loading (solid-phase concentration) on the adsorbent at the contact time *t*.

#### Kinetic Experiment

In order to investigate the different kinetics of the three cPEI adsorbents in more detail, a kinetic experiment was carried out for one selected PFAS (PFHxA), and the adsorption rates were compared with those of the TP108 IX. For this model solution, 10 μg/L PFHxA was prepared in four 5 L batches, from which 10 mL was taken for the determination of the respective reference concentration *c*_0_. Subsequently, 200 mg of adsorbent (dry weight) was soaked in 10 mL ultrapure water (Milli-Q) for 12 h and added to the remaining 4990 mL model solution, resulting in an adsorbent concentration of 40 mg/L. Prior soaking was conducted to ensure that the kinetics of the adsorbents were not limited by the previous drying. The dilution effect caused by the addition of 10 mL ultrapure water was negligible (0.2%).

Every batch was continuously stirred with glass-coated magnetic stirrers at 1100 rpm, and 6 mL samples were taken after 0.25, 0.5, 1, 4, 7, 24, and 48 h. Batch volume change caused by the sampling was negligible (<0.9%), the bottles were sealed with polypropylene screw caps during the experiment and only opened for sampling.

Reaction kinetic rate constants *k*_1_ and *k*_2_ were fitted using the pseudo-first order rate expression (PFO, eq 3) and pseudo-second order rate expression (PSO, eq 5) in its respective linearized form (eqs 4 and 6).

$$q(t)=q_{e}(1-e^{-k_{1}t})$$ 3

$$\ln\left(\frac{q_{e}-q(t)}{q_{e}}\right)=-k_{1}t$$ 4

$$q(t)=\frac{q_{e}^{2}k_{2}t}{1+q_{e}k_{2}t}$$ 5

$$\frac{t}{q(t)}=\frac{1}{q_{e}}t+\frac{1}{k_{2}q_{e}^{2}}$$ 6

The derivations of the PFO and PSO equations and a comprehensive theoretical background can be found in the publications of Revellame et al.^65^ and Ho and McKay.^66^ For the PFPE-cPEI and the TP108 IX, surface diffusion coefficients *D*_s_ were additionally determined using the freely available modeling software FAST (https://www.fast-software.de/).^67^ The homogeneous surface diffusion model (HSDM) was selected as the modeling approach, and the assumption was made that the film diffusion is negligible due to the high stirring velocity and that intraparticle diffusion is the rate-limiting step. The mass transport in the adsorbent particle is hereby described by Fick’s second law, where *r* is the radial coordinate of the adsorbent (eq 7):

$$\frac{\partial q}{\partial t}=D_{s}\left(\frac{\partial^{2}q}{\partial r^{2}}+\frac{2}{r}\frac{\partial q}{\partial r}\right)$$ 7

In order to examine the influence of neglecting film diffusion on the evaluation of the kinetics, the film diffusion coefficient *k*_L_ was also fitted in a second variant of the HSDM. For this, the assumption was made that the kinetics are dominated by film diffusion within the first 15 min of contact time. The film diffusion coefficient *k*_L_ was then determined using eq 8 as described by Worch et al.^68^ where *a*_m_ is the total adsorbent surface area related to the adsorbent mass (*m*_A_) available in the batchvolume (*V*_L_):

$$\ln\left(\frac{c}{c_{0}}\right)=-\frac{m_{A}}{V_{L}}k_{L}a_{m}t$$ 8

Subsequently, the diffusion coefficients were fitted as in the first variant but with an additional consideration of film diffusion. Freundlich isotherm data from our study was used to describe the adsorption equilibrium using the HSDM. A detailed theoretical background to the HSDM is described by Worch et al.^68^

#### Isotherm Experiment

Batches of 250 mL model solution contaminated with 10 μg/L TFMSA, PFPeS, PFBA, or PFHxA, respectively, were prepared in 500 mL glass bottles containing varying adsorbent concentrations (0, 10, 20, 30, 40, 50, 60, 70, and 80 mg/L). Samples were agitated for 48 h on a horizontal shaker (equilibrium) and sealed with polypropylene screw caps 48 h was selected as a sufficient equilibration time based on our own preliminary tests and is also in line with other studies^69,70^ that were carried out in comparable concentration ranges and under comparable conditions.

The isotherm model parameters *n*, *K*_F_, *K*_L_ and *q*_max_ were fitted using the Freundlich (eq 9) and Langmuir isotherm (eq 11) in its respective linearized form (eqs 10 and 12).

$$q=c^{n}K_{F}$$ 9

$$\log(q)=\log(c)n+\log(K_{F})$$ 10

$$q=q_{\max}\frac{K_{L}c}{1+K_{L}c}$$ 11

$$\frac{1}{q}=\frac{1}{c}\frac{1}{K_{L}q_{\max}}+\frac{1}{q_{\max}}$$ 12

#### PFAS Analysis

TFMSA was analyzed by a high-performance liquid chromatography coupled with tandem mass spectrometry (HPLC-MS/MS) method described in detail by Zeeshan et al.^71^ with a limit of quantification (LOQ) of 1 ng/L. PFHpA, PFOA, PFNA, PFDA, PFBS, PFHxS, and PFOS were analyzed following a HPLC-MS/MS method described in detail by Zietzschmann et al.,^72^ with a respective LOQ of 1 ng/L (PFHpA, PFOA, PFBS, and PFHxS) or 2 ng/L (PFNA, PFDA, and PFOS).

The concentrations of PFBA, PFHxA, and PFPeS after the adsorption experiments were determined using tandem mass spectrometric (Agilent 1290 infinity 2 Series HPLC coupled with SCIEX QTRAP 6500+ triple quadrupole mass spectrometer; LC-MS/MS) analysis. The chromatographic separation of these compounds was conducted on a Waters BEH C18 (100 mm × 2.1 mm × 1.7 μm) reverse phase column. A gradient elution of 0.1% acetic acid and pure methanol (flow rate: 0.25 mL/min; injection volume 20 μL) was used for this purpose. The compounds were analyzed in negative electrospray ionization (ESI) mode by monitoring the following ion transitions: PFBA (212.9 → 169.0), PFHxA (313.0 → 269.0), and linear PFPeS (349.0 → 80.0). The source parameters of LC-MS/MS are Curtain gas; 35 psi, Gas 1; 55 psi, Gas 2; 40 psi, Temperature; 450 °C, Ion spray voltage; −4000 V, and Entrance potential; −10 V. The compound-specific parameters used for LC-MS/MS analysis, such as the declustering potential, collision energy, and collision cell exit potential, were taken directly from literature (for PFBA and PFHxA^73^ and for linear PFPeS^74^). The respective LOQ values were 50 ng/L (PFBA and PFPeS) and 10 ng/L (PFHxA).

## Results and Discussion

The preparation of the three cPEI adsorbents followed three synthetic steps: cross-linking of bPEI (*M*_*w*_ = 10,000 g/mol),^75^ methylation with dimethyl sulfate, and ion exchange to chloride (Scheme S1). Diglycidyl ethers of the oligoethers PFPE (M = 522 g/mol), PEG (*M*_n_ = 500 g/mol),^76^ and PDMS (*M*_n_ = 800 g/mol)^77^ with similar molecular weights were selected as cross-linkers. The cross-linking with the PDMS cross-linker was noticeably slower than with the other two cross-linkers, and it took almost twice as long to obtain cPEI with a similar solid texture. The successful synthesis of the final cPEI adsorbents, PEG-cPEI, PDMS-cPEI and PFPE-cPEI (Chart 1) was confirmed by elemental analysis (EA) (Table S2), mercurimetric titration of chloride ions (Table S1), infrared (IR) spectroscopy (Figures 1a and S5) and X-ray photon (XP) survey spectra (Figure 1b and Table S3).

**Chart 1:**
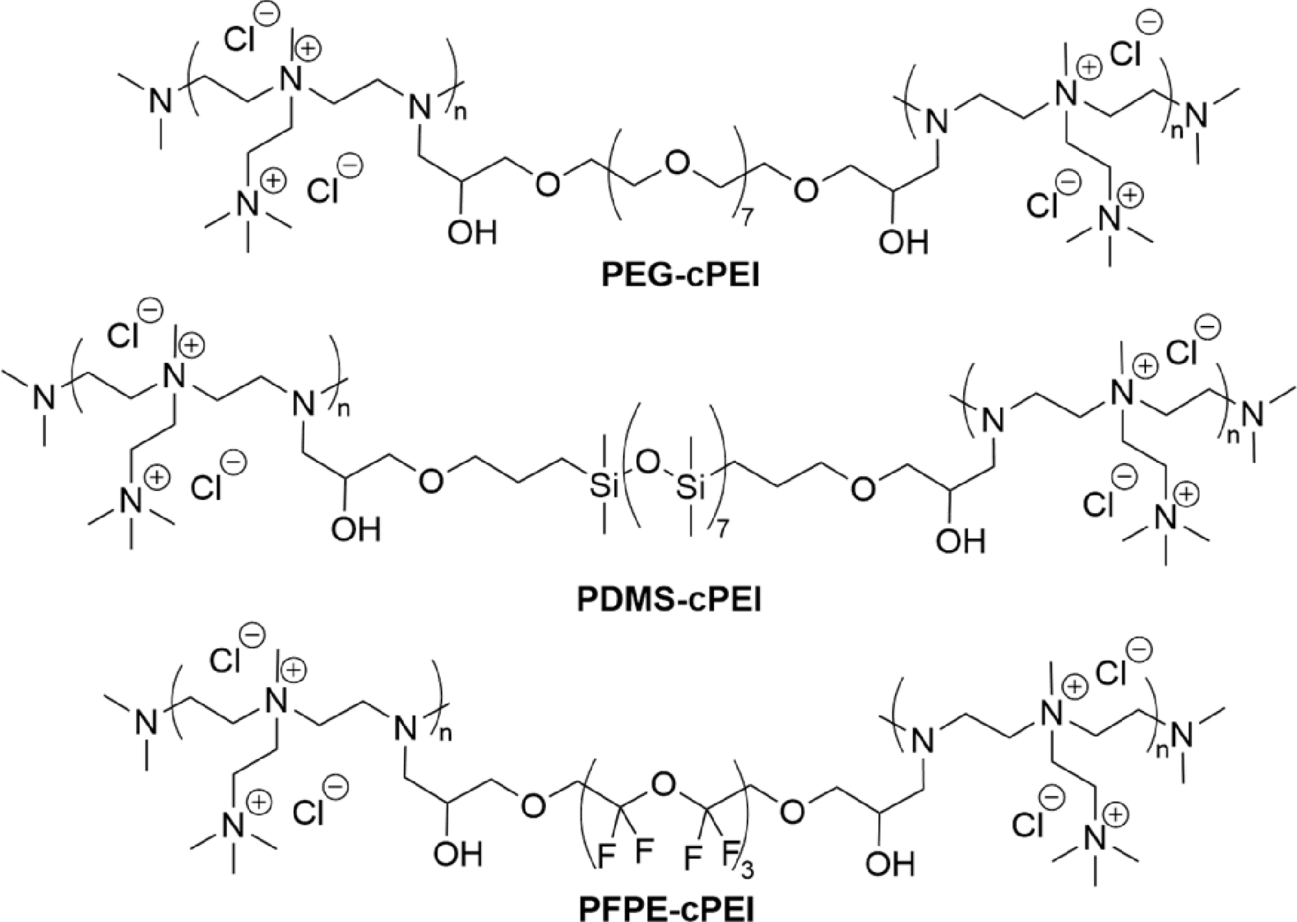
Chemical Structures of PEG-cPEI, PDMS-cPEI, and PFPE-cPEI

**Figure 1:**
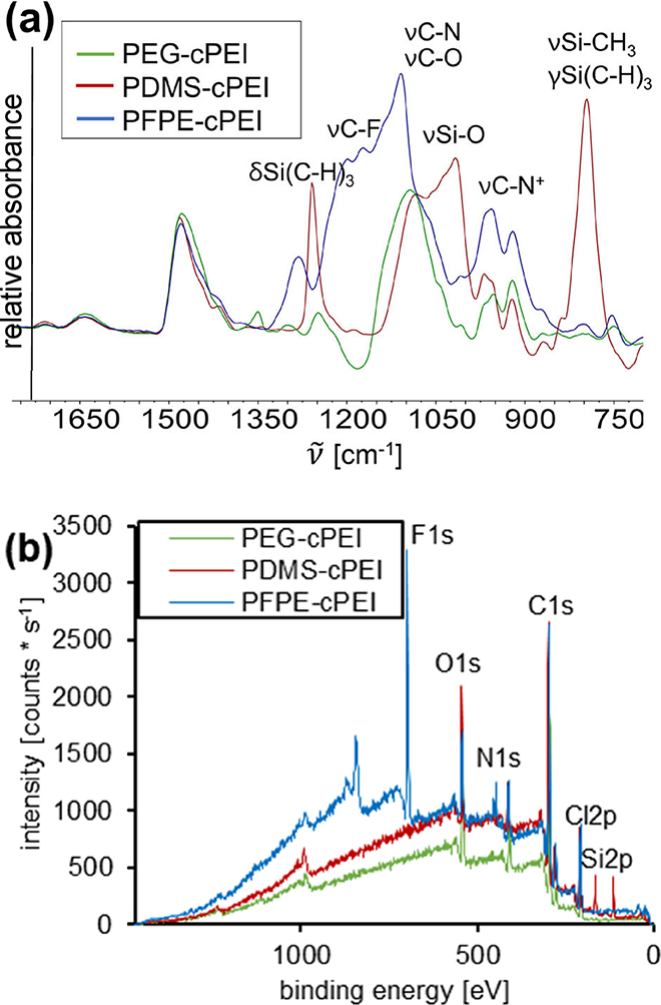
(a) IR absorption spectra of the cPEI adsorbents with marked bands of characteristic vibrations (full spectrum in Figure S5). (b) XP survey spectra of the cPEI adsorbents.

The IR spectra of all cPEI adsorbents show the expected stretching vibration bands of ether groups (∼1100 cm^–1^) and two bands that are commonly attributed to quaternary ammonium groups (∼960 and ∼920 cm^–1^).^24,34,78,79^ The IR spectrum of PFPE-cPEI further shows the broad, characteristic C–F stretching vibration band between 1240 and 1100 cm^–1^. Three typical bands for dimethyl siloxanes are detected at 1259, 1016, and 795 cm^–1^ in the IR spectrum of PDMS-cPEI.^80,81^ The quantification of the XP survey spectra (Table S3) shows chlorine to nitrogen ratios of 0.63:1 to 0.87:1, indicating a high degree of methylation despite the high charge repulsion of tightly packed ammonium groups in PEI.^9^ The cPEI adsorbents contain 4.4–4.9 mol/kg of chloride ions that are accessible for ion exchange (Table 1), corresponding to a share of quaternary nitrogen groups of roughly 70–75% (approximately 6.6 mol/kg chloride would be expected if all nitrogen atoms are quaternary ammonium groups with chloride counterion). The chloride content of TP108 IX is less than a third of that and also a bit lower than previously reported chloride contents (anion exchange capacities) of similar resins that were determined by different techniques.^40^ The cross-linker contents of the cPEI adsorbents were calculated from the decrease of the nitrogen contents as quantified by EA and the rise of the oxygen, silicon, or fluorine content in XP survey spectra. EA indicates cross-linker contents of 30–40 wt %, and XP survey spectra indicate cross-linker contents between 41 and 51 wt %. This proves that the cPEI adsorbents consist of a comparable amount of ionic as well as cross-linking groups, allowing us to establish correlations between the PFAS adsorption and the type of introduced cross-linker group.

**Table 1:** Contents of Cross-Linker in the Dried cPEI Adsorbents Calculated from the Nitrogen Content Determined by Elemental Analysis (EA) or from the Oxygen, Silicon, or Fluorine Content^a^

	cross-linker content			chloride content
sample	according to nitrogen (EA) [wt %]	according to oxygen (XPS) [wt %]	according to Si or F (XPS) [wt %]	according to titration [mol/kg]
TP108	/	/	/	1.26 ± 0.04
PEG-cPEI	40 ± 3	41.2 ± 0.9	/	4.36 ± 0.09
PDMS-cPEI	30 ± 2	49.8 ± 0.9	50.5 ± 1.1	4.94 ± 0.11
PFPE-cPEI	34 ± 1	46.1 ± 0.9	44.6 ± 0.7	4.62 ± 0.06

a The chloride content was determined by mercurimetric titration.

The cPEI adsorbents exhibit a wide range of morphologies, as can be seen in electron microscopic images (Figures S29–S31). The dry particles that were retained between two analytical sieves were collected (mesh sizes: 63 and 710 μm). This step was added to homogenize the adsorbent particles in this size range, which was complicated due to hygroscopically induced agglomeration. Optical microscopic images were used to evaluate the particle size distributions of the cPEI adsorbents in water after sieving (Figures 2 and S18–S25). Their median projected area diameters were 52 μm (PDMS-cPEI), 44 μm (PFPE-cPEI), and 26 μm (PEG-cPEI), and their median Feret diameters were 77, 62 and 37 μm, respectively. While the cPEI adsorbents showed similar particle size distributions, the TP108 IX consists of much larger particles (approximately 700 μm determined after sieving as described above). The cPEI adsorbents were also checked for porosity due to profound swelling of the cPEI adsorbents in water. However, neither SEM (Figures S29–S31, SEM of TP108: Figure S32) or Brunauer, Emmett, Teller (BET) surface area determination (PEG-cPEI: (0.072 ± 0.002) m^2^/g, PDMS-cPEI: (0.113 ± 0.002) m^2^/g, PFPE-cPEI: (0.076 ± 0.002) m^2^/g) revealed any types of pores. Similarly, small-angle X-ray scattering (SAXS) (Figure S1) did not yield a spectrum that would be expected for highly porous materials. Only PDMS-cPEI contains scatterers in the small nanometer range (Figure S2). Nevertheless, given the extremely small surface area, it is unlikely that this scattering originates from pores. The characterization of the adsorbents was completed with a study of their thermal stability using thermogravimetry. The three cPEI adsorbents are stable up to 200 °C in a nitrogen atmosphere, so they are thermally less stable than pristine bPEI (start of degradation around 290 °C) but more stable than TP108 IX (start of degradation around 160 °C) (Figure S3).

**Figure 2:**
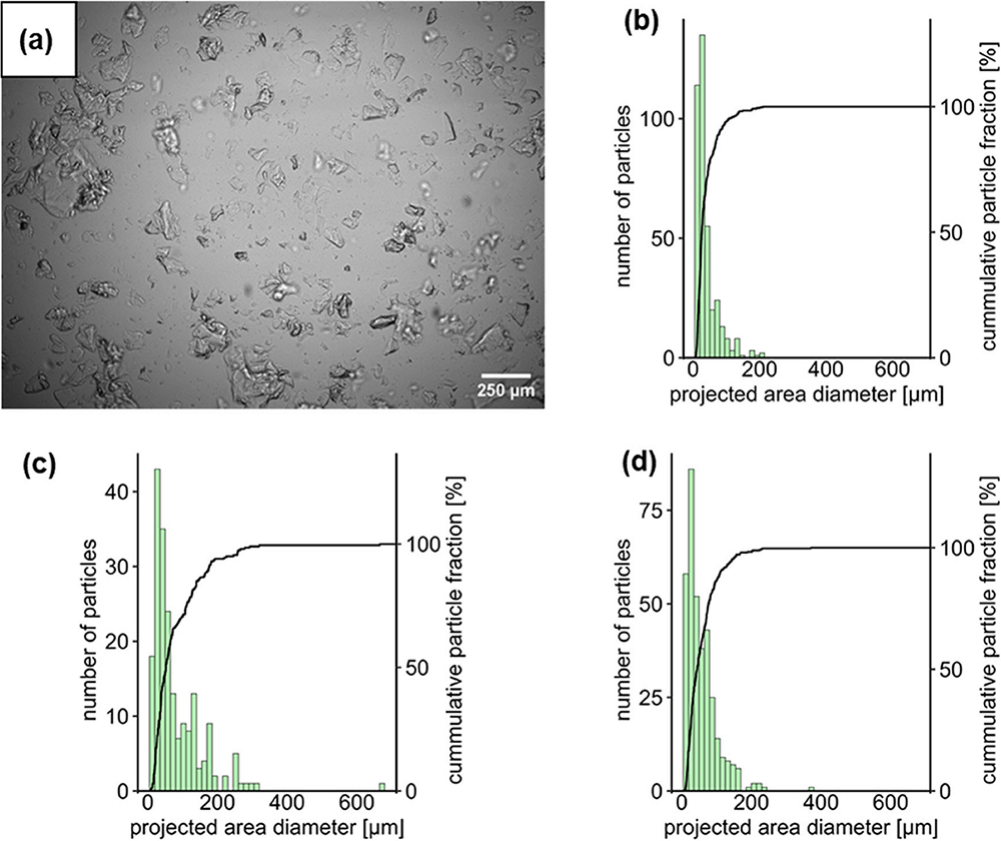
(a) Optical microscopic image of PFPE-cPEI. Number-weighted histograms including cumulative curves of the size distribution of the projected area diameters of (b) PEG-cPEI (*n* = 387), (c) PDMS-cPEI (*n* = 200), and (d) PFPE-cPEI (*n* = 353) particles in optical microscopic images.

Screening experiments revealed that all adsorbents were able to remove the PFAS investigated, albeit with different kinetics (Figure 3). While the three cPEI adsorbents were able to achieve more than 70% of the maximum loading for all PFAS after just 30 min, the IX and especially the GAC showed significantly slower kinetics. The slow kinetics of GAC can be attributed to slow diffusion along the surface and within the pores of the highly porous adsorbent.^82^

**Figure 3:**
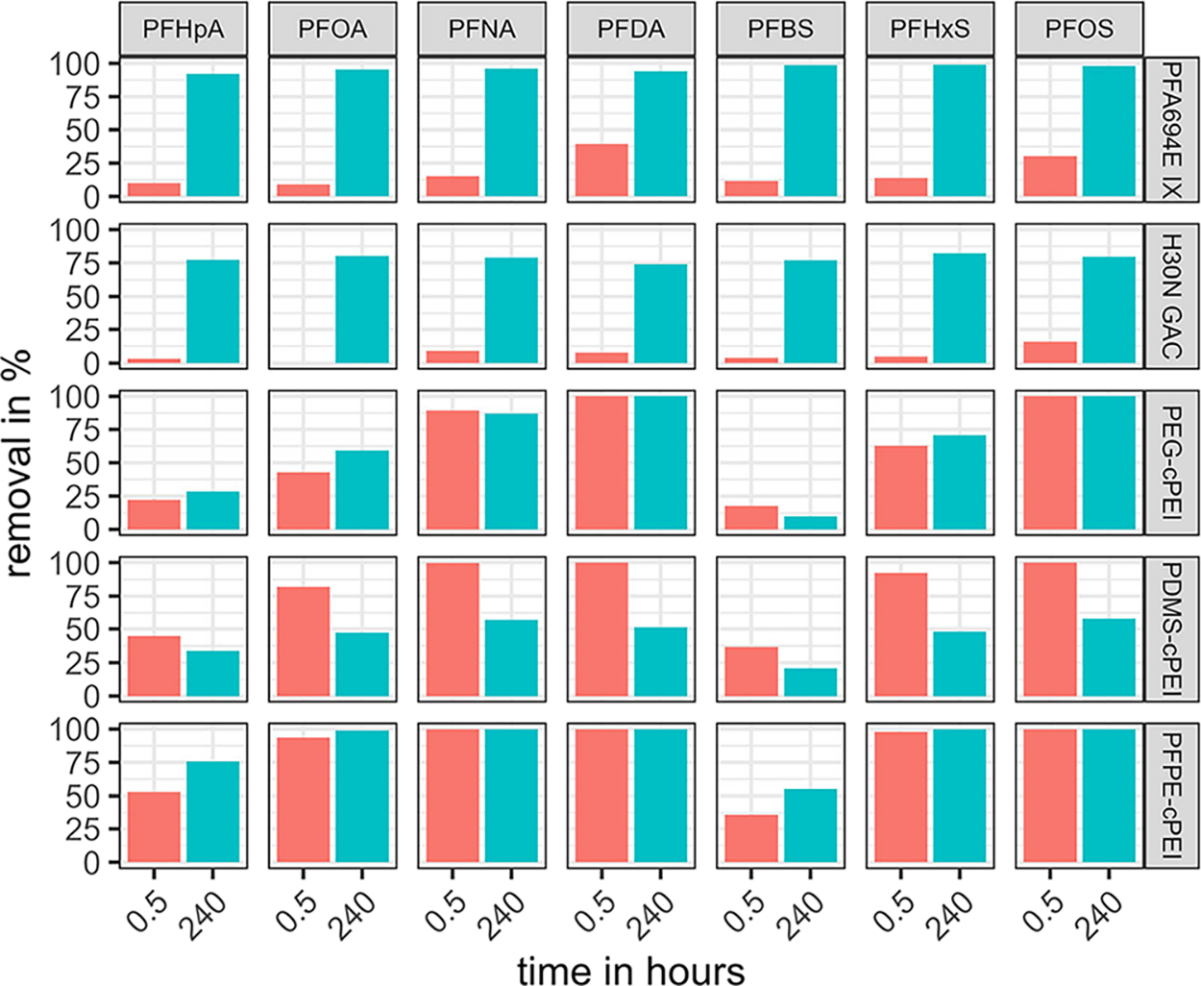
Percentage removals of PFSA and PFCA (with different numbers of perfluorinated carbon atoms) after 30 min and 10 days at an adsorbent dose of 40 mg/L (dry weight) for the commercial PFA694E IX and H30N GAC and the newly synthesized PEG-cPEI, PDMS-cPEI, and PFPE-cPEI. Reference concentrations were 6.4 μg/L PFHpA, 8.9 μg/L PFOA, 6.3 μg/L PFNA, 2.8 μg/L PFDA, 6.6 μg/L PFBS, 8.0 μg/L PFHxS, and 4.9 μg/L PFOS.

In the case of the commercial PFA694E IX, only slight differences in the loadings of different PFAS could be determined at equilibrium due to the high removal observed for all PFAS considered; it proved to be the most effective adsorbent, particularly for the short-chain compound PFBS. PFA694E was used solely for this screening as a reference because of a change of supplier during the course of the study. However, our own studies indicate that only minor differences between the PFAS-specific resins of the various manufacturers are to be expected.^83^ Unexpectedly, GAC showed only slight differences in the removal of PFAS of different chain lengths. Other studies have reported a stronger influence of the PFAS chain length on the removability by GAC.^7,84^

All cPEI adsorbents were able to remove long-chain PFAS better than PFAS with shorter chains (e.g., *K*_d,PFHpA_ = 0.01 L/kg and *K*_d,PFDA_ = 8.60 L/kg for the PEG-cPEI). With the same number of perfluorinated carbon atoms, it was also observed that PFSA could be removed better than PFCA with all adsorbents (e.g., *K*_d,PFNA_ = 0.17 L/kg and *K*_d,PFOS_ = 23.23 L/kg for the PEG-cPEI).

Among the cPEI adsorbents, PFPE-cPEI achieved the highest removals. With a comparable anion exchange capacity (see Table 1), the differences here can be attributed to the different cross-linkers. The hydrophobic interactions between the perfluorinated cross-linkers and the perfluorinated carbon tail of the PFAS as well as electrostatic attractions between the deprotonated acid group and the quaternized ammonium group on the PFPE-cPEI can be identified as governing adsorption mechanisms.^85^

The PDMS-cPEI showed removals comparable to that of PFPE-cPEI after 30 min, but the removals were decreased after 10 days, which was further investigated in additional kinetic studies.

The kinetics of cPEI adsorbents and selected IX (TP108) were investigated in more detail (Figure 4). The fitting of the two reaction kinetic models, PFO and PSO, showed that the PSO provided a better mathematical approximation of the kinetic curves for all four adsorbents (compare root-mean-square error (RMSE) in Table 2). However, simplified conclusions about the adsorption mechanism and rate controlling step should not be drawn from this model, as discussed in more detail in several publications.^68,86,87^ The poorer mathematical fit of the PFO could also be linked to the experiment design: the cPEI adsorbents already reach their equilibrium loading within the first few hours of the experiment; the left-hand side of eq 4 is no longer defined when *q*(*t*) approaches *q*_e_. A higher sampling resolution before reaching equilibrium could therefore influence the evaluation of the models and favor the PFO model. Nevertheless, the PSO allows for a quantification of the adsorption rate and should be rather understood as an empirical equation in the described context of this study. The kinetic rate constants *k*_2_ of the cPEI adsorbents were significantly higher than those of the TP108 IX (e.g., approximately 2000 times higher for the PFPE-cPEI). In practice, faster kinetics mean shorter contact times and thus smaller required reactors.

**Figure 4:**
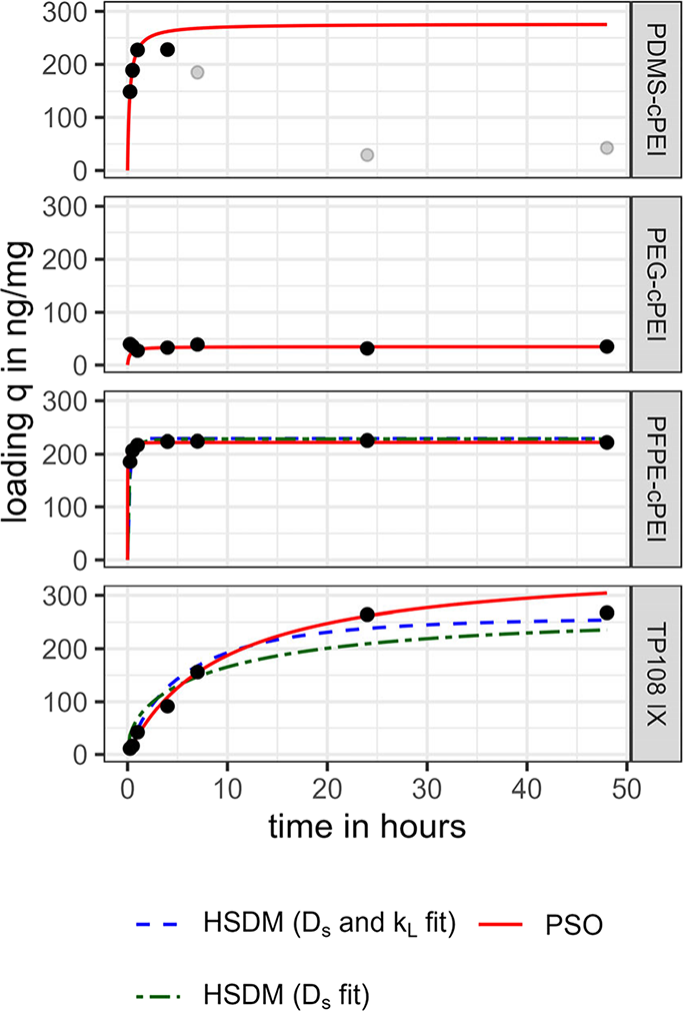
PFHxA loading at 40 mg/L adsorbent dose over 48 h with PSO model for all adsorbents and HSDM for PFPE-cPEI and TP108. PFHxA reference concentrations were 10.9 μg/L (TP108), 10.4 μg/L (PEG-cPEI), 10.0 μg/L (PDMS-cPEI), and 10.5 μg/L (PFPE-cPEI). All samples were analyzed in duplicates. Data points at contact times above 4 h were neglected for PDMS-cPEI model fitting (neglected data points in gray).

**Table 2:** Adsorption Kinetic Rate Constants for PFO and PSO Model with Respective Coefficient of Determination *R* ^2^ for Linearized Model and RMSE for Model Curve^a^

	PFO				PSO			
adsorbent	*k*_1_ [1/h]	*q*_e_ [ng/mg]	*R* ^2^	RMSE [ng/mg]	*k*_2_ [mg/(ng h)]	*q*_e_ [ng/mg]	*R* ^2^	RMSE [ng/mg]
TP108	0.1835	267.4	0.9886	23.1	0.0003	364.3	0.9680	15.7
PEG-cPEI	–0.0017	40.0	0.0014	35.7	0.1407	34.6	0.9965	9.3
PDMS-cPEI	6.6491	227.9	0.9619	27.0	0.0164	276.5	0.9994	19.8
PFPE-cPEI	0.0247	225.5	0.1143	176.0	0.6071	222.3	1.0000	12.6

a Determined for PFHxA at 40 mg/L adsorbent dose (compare with Figure 4).

Experimentally determined diffusion coefficients have a higher informative value for adsorption mechanisms than the kinetic rate constants^86,87^ and can serve as input parameters for further adsorption process modeling.^68^ However, the calculation of surface diffusion coefficients *D*_s_ requires a higher computational effort and the additional knowledge of isotherm data.^68^ A *D*_s_ of 7 × 10^–15^ m^2^/s was determined for the PFPE-cPEI (RMSE = 4.2 ng/mg) and a *D*_s_ of 3 × 10^–14^ m^2^/s (RMSE = 32.1 ng/mg) for the TP108 IX, assuming negligible film diffusion. With simultaneous consideration of film and surface diffusion, a *D*_s_ of 7 × 10^–14^ m^2^/s and a *k*_L_ of 1 × 10^–4^ m^2^/s were determined for the PFPE-cPEI (RMSE = 12.6 ng/mg) and a *D*_s_ of 9 × 10^–14^ m^2^/s and a *k*_L_ of 8 × 10^–5^ m/s (RMSE = 18.8 ng/mg) for the TP108 IX. The improved model fit indicates that, at least in the case of TP108 IX, film diffusion does not appear to be completely negligible. In both variants, however, faster or comparable intraparticle mass transport was determined for TP108 IX compared to the PFPE-cPEI.

One of the key differences between the PSO and the HSDM is that the driving force of the PSO is the difference between the (constant) mean equilibrium loading and the loading at time *t*, whereas the HSDM accounts for the concentration gradient within the particle.^68^ When using the HSDM, the particle size is directly included in the calculation.

The results therefore indicate that the explanation for the fast adsorption by the cPEI adsorbents is not rapid intraparticle diffusion, but rather the small particle sizes (52 μm (PDMS-cPEI), 44 μm (PFPE-cPEI), 26 μm (PEG-cPEI) vs 700 μm (TP 108)) and associated larger outer surface areas (which also favors faster film diffusion, compare eq 8) as well as directly accessible adsorption sites.

The kinetic curve of the PDMS-cPEI showed that the PFHxA concentration increased again after about 4 h. This phenomenon could occur due to reversible binding of PFHxA or instability of the PDMS-cPEI adsorbent. PDMS is known for its great chemical stability against many chemicals, but it is readily degraded by fluoride ions because of the high stability of Si–F bonds.^88^ A measurement of the reference sample adhering to DIN EN ISO 10304-1 (D20)^89^ ruled out an increased fluoride concentration (*c*_fluoride_ < 0.1 mg/L). A degradative fluoridation of the PDMS cross-linker seems, therefore, unlikely as an explanation for the elevated PFHxA concentrations. Displacement of PFHxA at the adsorption sites by competing substances also appears unlikely, since a model solution with only NaHCO_3_ and NaCl in addition to PFHxA was used, and similar displacement effects should be visible for the other cPEI adsorbents. However, stirring the solutions or agitation on the horizontal shaker might have pulverized the potentially mechanically less stable PDMS-cPEI particles. As the initial removal within the first hours looks promising, it would be advisable to further investigate the underlying mechanisms for reversible PFHxA removal by the PDMS-cPEI.

Due to the reversible PFHxA removal observed in the kinetic experiment after 4 h (compare Figure 4), the PDMS-cPEI was omitted from further isotherm studies. The PEG-cPEI showed significantly lower removals than the PFPE-cPEI, which is why no removal could be observed for the investigated short-chain PFAS at low adsorbent doses. The presentation and fit of isotherm models are therefore omitted, and only single-point adsorption coefficients at the highest adsorbent dose (80 mg/L) were discussed for comparison.

Both the Freundlich and Langmuir isotherm models can describe the isotherm curves of the TP108 IX and the PFPE-cPEI (compare Figure 5). However, the Freundlich model appears to give a better fit, as can be seen from a direct comparison of the RMSE values in Tables 3 and 4. Both Freundlich and Langmuir are single-solute isotherm models.^68^ When using adsorbents in which ion exchange contributes to the removal, such as for TP108 IX and cPEI adsorbents with quaternized ammonium groups, it is not correct to consider a single solute system, as the PFAS compete with the counterion with which the ion exchanger is loaded (in this case, chloride). The application of the models here should therefore be understood as empirical, and the parameters determined are only valid under the experimental conditions described, as discussed in detail by Haupert et al.^90^ The smaller the Freundlich exponent *n* is, the more concave the isotherm shape is, yielding high loadings at low concentrations. Favorable isotherms can be observed for the TP108 IX, which (with the exception of PFPeS) shows a higher loading than the PFPE-cPEI at the higher adsorbent doses. The isotherms of the PFPE-cPEI in the tested concentration ranges revealed almost linear shapes with Freundlich exponents close to 1 (except for PFBA).

**Figure 5:**
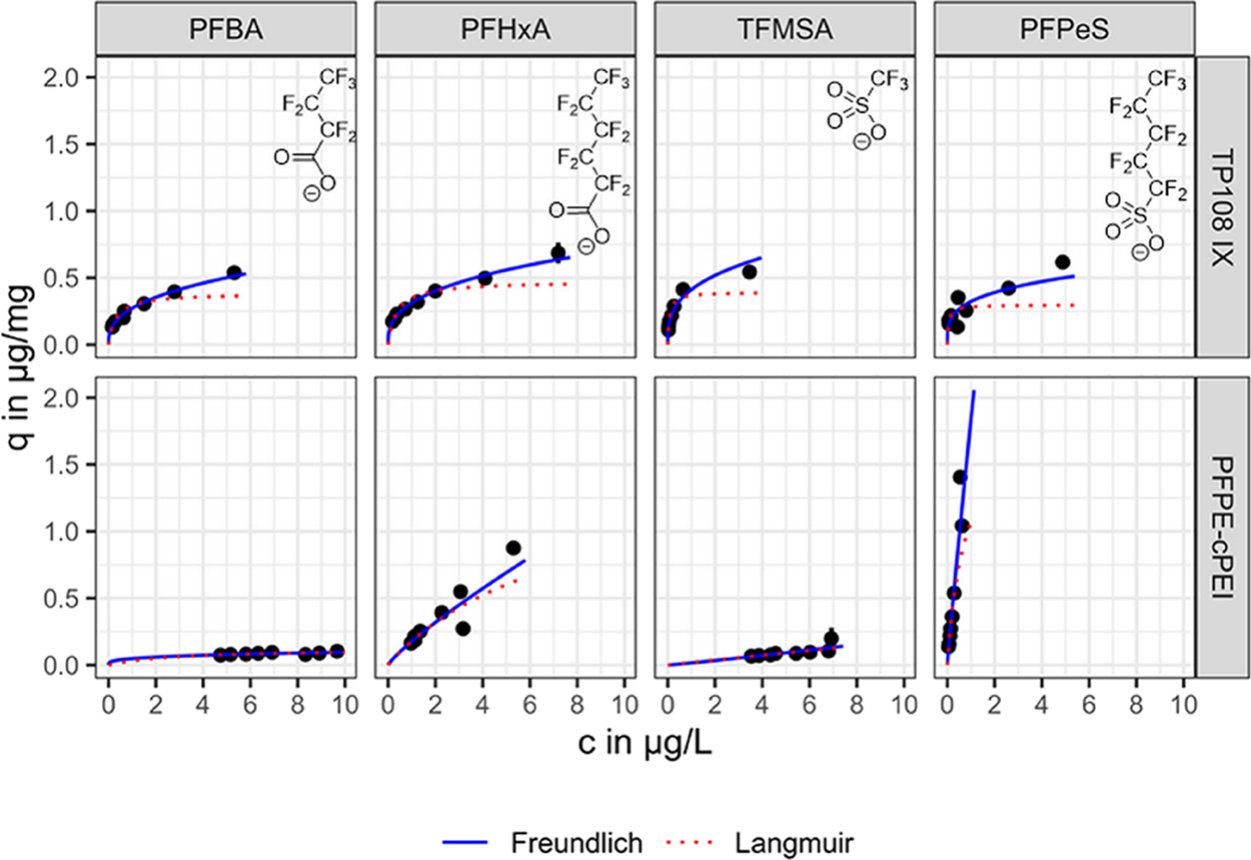
Isotherm data after 48 h equilibration with respective modeled isotherms according to Freundlich and Langmuir for PFPE-cPEI and TP108 IX. Reference concentrations were 12.9 μg/L PFBA, 13.5 μg/L PFHxA, 8.4 μg/L TFMSA, and 11.3 μg/L PFPeS. All samples were analyzed in duplicates. The structures of the anions of the PFCA and PFSA are displayed in the top right corners.

**Table 3:** Freundlich Model Parameters with Respective Coefficient of Determination *R* ^2^ for the Linearized Model and RMSE for the Model Curve

adsorbent	PFAS	*n*	*K*_F_ [ng/mg/(ng/L)^*n*^]	*R* ^2^	RMSE [ng/mg]
PFPE-cPEI	PFBA	0.30	6.2370	0.5093	6.3
	PFHxA	0.84	0.5245	0.8263	95.3
	TFMSA	1.09	0.0085	0.6704	26.4
	PFPeS	0.94	2.7544	0.9689	56.1
TP108	PFBA	0.38	20.1674	0.9831	15.6
	PFHxA	0.36	25.8348	0.9902	20.7
	TFMSA	0.33	41.6775	0.9671	35.5
	PFPeS	0.27	48.5026	0.6703	71.8

**Table 4:** Langmuir Model Parameters with Respective Coefficient of Determination *R* ^2^ for Linearized Model and RMSE for Model Curve

adsorbent	PFAS	*q*_max_ [ng/mg]	*K*_L_ [L/ng]	*R* ^2^	RMSE [ng/mg]
PFPE-cPEI	PFBA	121.5	3.57 × 10^–04^	0.5261	6.3
	PFHxA	1486.7	1.36 × 10^–04^	0.8505	117.4
	TFMSA	1746.4	1.10 × 10^–05^	0.7743	27.9
	PFPeS	1853.7	1.35 × 10^–03^	0.9648	69.9
TP108	PFBA	384.6	3.29 × 10^–03^	0.9140	67.7366
	PFHxA	473.4	2.81 × 10^–03^	0.9024	89.5204
	TFMSA	393.4	1.42 × 10^–02^	0.9460	63.8401
	PFPeS	297.9	1.81 × 10^–02^	0.3138	135.7011

For similar Freundlich exponents, the affinities of the adsorbates to the adsorbent can be compared according to the respective *K*_F_ values. The estimate for the TP108 IX results in the following sequence: *K*_F,PFPeS_ > *K*_F,TFMSA_ > *K*_F,PFHxA_ > *K*_F,PFBA_. The higher affinity of TFMSA (compared to PFHxA) indicates that the functional anionic group of the molecule has a stronger influence on the removal of short-chain PFAS by the TP108 IX than the length of the perfluorinated carbon chain. The removal here is primarily dominated by electrostatic attraction. The results are different in the case of PFPE-cPEI, which yielded very low removals for the shortest chain PFAS, TFMSA, and PFBA. In contrast, in the case of the most hydrophobic compound PFPeS, the PFPE-cPEI achieved higher loadings than the TP108 IX. With the PEG-cPEI, removals of a similar order of magnitude could not be achieved for any short-chain PFAS. While the *K*_d_ values at 80 mg/L adsorbent for TFMSA were about 6 times lower, those for PFPeS were even about 150 times lower than those of the PFPE-cPEI (compare *K*_d_ values in Table S5).

It can be concluded that the presence of the fluorous PFPE cross-linker enhances the capacity for most PFAS investigated drastically. The poorer removal of the shortest-chain substances (PFBA and TFMSA) with simultaneously comparable or even higher removal of PFAS with at least 5 perfluorinated C atoms (compare Figures 3 and 5) indicates that hydrophobic interactions play a more important role during the adsorption of PFAS onto the PFPE-cPEI than onto the TP108 IX.

## Conclusions

Three types of cross-linked PEI resin particles (cPEI) were synthesized, characterized in detail, and evaluated regarding their potential as adsorbents for anionic PFAS in comparison to an industrial state-of-the-art IX for PFAS removal. The three cPEI adsorbents differed in their oligoether cross-linker segment (PFPE, PDMS, or PEG) but showed comparable anion exchange capacities, cross-linker contents, surface areas, and morphologies. All investigated cPEI adsorbents showed significant long-chain PFAS removal efficiencies within only 30 min. In contrast, PFAS were only poorly removed by commercial IX and GAC in the same time span. The fast adsorption rate onto the cPEI adsorbents is reflected by the reaction kinetic constants for the adsorption of one selected PFAS (PFHxA). The kinetics of all adsorbents could be described well using the PSO model. Nevertheless, diffusion coefficients that were calculated using the HSDM indicated that intraparticle diffusion of PFHxA in the state-of-the-art IX was faster than in the PFPE-cPEI. The cPEI adsorbents preferably adsorbed PFAS with longer chains, while the commercial IX obtained more consistent removals of PFAS regardless of their chain lengths. This observation was confirmed with isotherm studies for PFHxA, PFBA, TFMSA, and PFPeS and the adsorbents PFPE-cPEI and the commercial IX. It should be emphasized that the adsorption isotherms provide only an empirical description of the adsorption process and are limited to the conditions used in our test. Overall, our results indicate that hydrophobic cross-linked PEI is a promising type of adsorbent for the industrial-scale remediation of PFAS when fast kinetics are required. The PDMS-cPEI is a promising fluorine-free adsorbent for PFAS removal with very short contact times. However, in order to support large-scale applications of PDMS-cPEI, further research is necessary to ensure its stability during longer operation.

## Supplementary Material



## Notes

Experimental descriptions of the mercurimetric titration, thermogravimetric analysis, ESI mass spectrometry, NMR, EA, IR, XPS, SEM, SAXS, BET, as well as calculations, microscopic images, histograms, and single-point adsorption coefficients (PDF)
