# Supplementary material for: Effect of Hydrophobic Cross-Linkers in Strong Base Gel-Type Resins on the Adsorption Kinetics and Capacity for Perfluoroalkyl Substances
Source: ACS ES T Water. 2025 Jun 13;5(8):4435–47. doi: 10.1021/acsestwater.5c00094 (PMC12340944; doi:10.1021/acsestwater.5c00094)
Supplement: Supplementary file 1 [file ew5c00094_si_001.pdf]

Effect of Hydrophobic Cross-Linkers in Strong Base Gel-Type Resins on the Adsorption Kinetics and Capacity for Perfluoroalkyl Substances

Florian Junge,[‡] Fiona E. Rückbeil,[‡] Regina Gnirss, Rainer Haag, Alejandro Lorente, Fabio Lorenz, Sunil P. M. Menacherry. Aki S. Ruhl, Alexander Sperlich, Ana Zidar, Olaf Wagner**

[‡]Both authors contributed equally to this work.

Institut für Chemie und Biochemie, Freie Universität Berlin, Arnimallee 22, 14195 Berlin, Germany

E-mail: olaf.wagner@fu-berlin.de

Technische Universität Berlin, Water Treatment, KF4, Fasanenstraße 1A, 10623 Berlin, Germany

E-mail: aki.s.ruhl@tu-berlin.de

Table of Contents

Chloride Quantification by Mercurimetric Titration.. 3
Calculation of the Mass Fraction of the Cross-Linker in the Adsorbents 4
Thermogravimetric Analysis.. 8
Electron Spray Ionization Time of Flight Mass Spectrometry (ESI-ToF-MS)................................... 8

Nuclear Magnetic Resonance 8
CHN Elemental Analysis 9
Fourier Transform Infrared Spectroscopy 10
Surface Area Determination Using BET Krypton Adsorption 10
Scanning Electron Microscopy 10
X-ray Photon Spectroscopy 10
Infrared Spectra 12
NMR Spectra 13
Mass Spectra 14
BET Isotherms 16
Light Microscopic Images (selection) 19
Histograms of the Particle Size Distribution 23
Scanning Electron Microscopic (SEM) Images 26
Single Point Adsorption Coefficients 32
Synthesis Scheme 3
References 33

Synthesis Scheme

Scheme S1. Preparation of the adsorbents from bPEI using PEG-, PDMS- and PFPE-based cross-linkers.

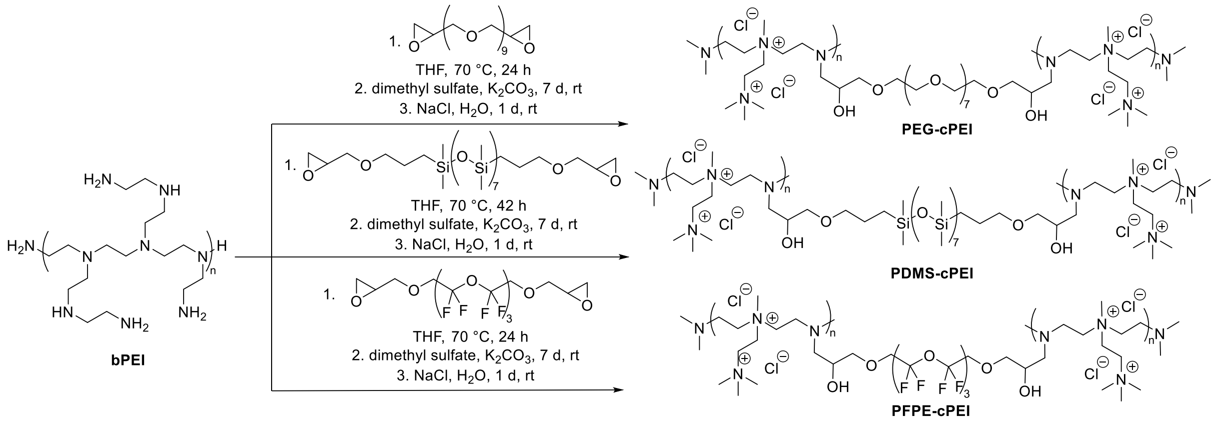

Chloride Quantification by Mercurimetric Titration

The amount of chloride ions in the adsorbents was quantified in dublicate using a MQuant Chloride Test kit 1.11106.0001 from Supelco (Merck KGaA). The titration was performed according to the standard procedure of the test kit. [1] (10.0 ± 0.3) mg of the respective adsorbent were diluted with MilliQ water (5.00 mL). The suspension turned blue after the addition of two drops of a solution of the indicator 1,5-diphenyl carbazone in *N,N'*-dimethyl acetamide. Afterwards, the aqueous suspension with the adsorbents was acidified using nitric acid solution till the colour became yellow. The yellow suspension was titrated with mercury (II) nitrate solution. The equivalence point is indicated by a colour change from yellow to violet. The concentration of chloride ions in mg/L c_{Cl^-} is obtained by reading the scale of the titration pipette at the equivalence point. The amount of chloride ions Cl^- in mol/kg was calculated using eqs 1 and 2.

$$Cl^- = \frac{V \cdot c_{Cl^-}}{M_{Cl} \cdot m_{adsorber}} \tag{1}$$

$$Cl^- \left[\frac{\text{mol}}{\text{kg}}\right] = \frac{5 \cdot c_{Cl^-}\left[\frac{\text{mg}}{\text{L}}\right]}{35.453 \cdot m_{adsorber}[\text{mg}]} \tag{2}$$

Table S1 Results of the chloride titration test.

sample	weight [mg]	Cl^- [mg/L]	Cl^- [mmol/g]
TP108	9.8, 9.8	86, 90	1.24, 1.29
uPEG-cPEI	10.1, 10.1	22, 10	0.31, 0.14
PEG-cPEI	9.9, 10.0	302, 314	4.30, 4.42
uPDMS-cPEI	9.8, 9.9	0, 0	0, 0
PDMS-cPEI	10.2, 10.0	352, 356	4.86, 5.01
uPFPE-cPEI	10.0, 10.2	0, 0	0, 0
PFPE-cPEI	9.9, 10.2	322, 338	4.58, 4.67

Calculation of the Mass Fraction of the Cross-Linker in the Adsorbents

The mass fraction of the cross-linker w_{cross} was calculated using elemental analysis and XP survey spectra. The nitrogen mass fraction w_N of the intermediate after cross-linking and of the final product after quaternization were measured by elemental analysis and are related to w_{cross}

by eqs 3 and 4. The underlying principle is, that the nitrogen of the intermediate is solely dependent on the PEI content. Upon quaternization, the cross-linker to nitrogen ratio is constant, as neither nitrogen nor further cross-linker is introduced or eliminated. Using the theoretical nitrogen content of PEI (32.52 %) and assuming the nitrogen content of the cross-linker to be 0 in reality (despite the traces detected in elemental analysis, as these are presumably minor impurities that are not part of the cross-linker molecule), one can obtain eq 5. This equation was finally used to compute w_{cross} and its uncertainty using the Gaussian method of the NIST uncertainty machine (version 1.6.2).[2, 3]

$$w_{cross,intermediate} = \frac{w_{N,intermediate} - w_{N,PEI}}{w_{N,crosslinker} - w_{N,PEI}} \tag{1}$$

$$w_{cross} = \frac{w_{N,product}}{w_{N,intermediate}} w_{cross,intermediate} \tag{2}$$

$$w_{cross} = w_{N,product}\left(\frac{1}{w_{N,intermediate}} - \frac{1}{0.3252}\right) \tag{3}$$

The XP survey spectra directly yielded the mole fraction (χ) of the elements. As oxygen (and for PDMS-cPEI additionally silicon and for PFPE-cPEI additionally fluorine) is solely present in the cross-linker and nitrogen is solely present in the PEI part, the mass fraction can be calculated using eqs 6 to 8. The molar mass of the PEI repeating unit was estimated using a weighted average from the molar mass of the sum formulas of the quaternary ($C_4H_{10}ClN$) and tertiary (C_3H_7N) PEI repeating unit (eq 9). This is based on the approximation of no remaining primary or secondary amines as well as omitting the contribution of the cross-linker to the alkylation of amines (and only considering the methyl groups introduced in the methylation step). Equation 10 was finally used to compute w_{cross} and its uncertainty using the Gaussian method of the NIST uncertainty machine (version 1.6.2).[2, 3] As a control for PDMS-cPEI and PFPE-cPEI, the number of oxygen atoms and χ_O were exchanged with the number of silicon/fluorine atoms and χ_{Si} and χ_F respectively in an additional calculation. The following values were used: PFPE-cPEI: M_{cross} = 522.2 g/mol, 7 oxygen atoms, 12 fluorine atoms; PDMS-cPEI: M_{cross} = 807.5 g/mol, 11 oxygen atoms, 8 silicon atoms; PEG-cPEI: M_{cross} = 504.6 g/mol, 11.5 oxygen atoms (since there seem to be 9 to 10 repeating units of PEG in the cross-linker according to the molecular weight in the TDS and ESI-ToF-MS).

$$w_{cross} = \frac{\chi_{cross} \cdot M_{cross}}{\chi_{cross} \cdot M_{cross} + \chi_{PEI} \cdot M_{PEI}} \quad (6)$$

$$\chi_{PEI} = \frac{\chi_N}{number\ of\ nitrogen\ atoms\ per\ PEI\ repeating\ unit} \quad (7)$$

$$\chi_{PEI} = \frac{\chi_N}{number\ of\ nitrogen\ atoms\ per\ PEI\ repeating\ unit} \quad (8)$$

$$M_{PEI} \approx \frac{\chi_{Cl}}{\chi_N} \cdot 107.58 \frac{\mathrm{g}}{\mathrm{mol}} + \left(1 - \frac{\chi_{Cl}}{\chi_N}\right) \cdot 57.10 \frac{\mathrm{g}}{\mathrm{mol}} \quad (9)$$

$$w_{cross} = \frac{1}{1 + \frac{number\ of\ oxygen\ atoms\ per\ crosslinker\ molecule}{\chi_O \cdot M_{cross}} \cdot \left(\chi_{Cl} \cdot 50.48 \frac{\mathrm{g}}{\mathrm{mol}} + \chi_N \cdot 57.10 \frac{\mathrm{g}}{\mathrm{mol}}\right)} \quad (10)$$

Small-angle X-ray Scattering (SAXS)

Small-angle X-ray scattering (SAXS) was measured using the MOUSE (Methodology Optimization for Ultrafine Structure Exploration) [4] set up equipped with an in-vacuum Eiger 1M detector (Dectris, Switzerland), which was placed at multiple distances between 55 mm to 2507 mm from the sample. Microfocus X-ray tubes were used to generate X-rays. The X-ray beams were parallelized and monochromatized to wavelengths of Cu Kα (λ = 0.154 nm) and Mo Kα (λ = 0.711 nm) by multilayer optics. A standardized complete 2D correction pipeline with uncertainty propagation was used to process the resulting data and to scale it to absolute intensity using the DAWN software package. [5, 6] The data was further fitted and analyzed using McSAS, a Monte Carlo method to extract form-free size distributions. [7]

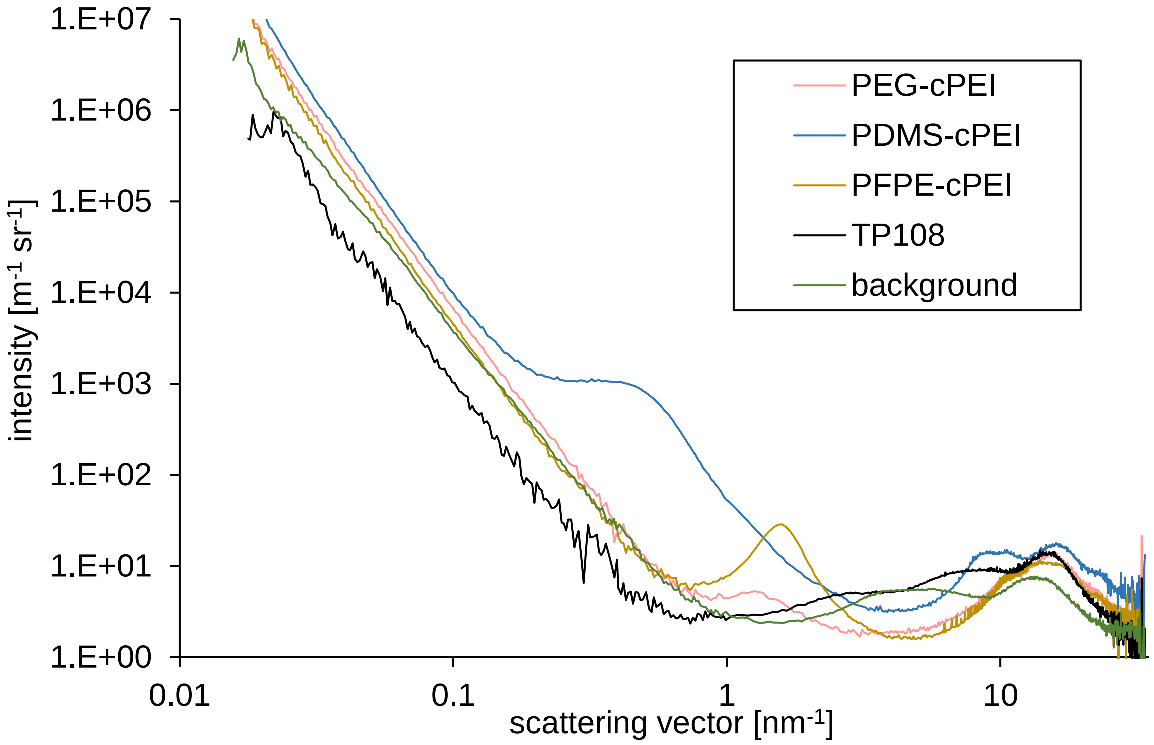

Figure S1. SAXS spectrum of PEG-cPEI, PDMS-cPEI, PFPE-cPEI and TP108.

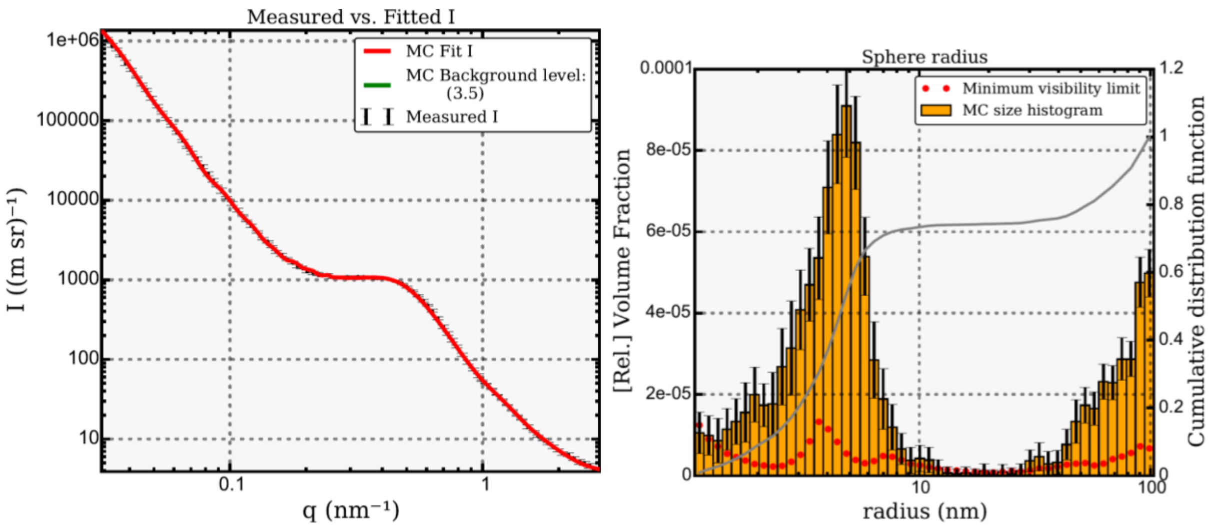

Figure S2. Fitted SAXS spectrum of PDMS-cPEI (left) and the volume-weighted size distribution of scattering phases in the same sample (right).

Thermogravimetric Analysis

Thermogravimetry was measured on a TGA 8000 from PerkinElmer (Rodgau, Germany) under nitrogen atmosphere. A few milligrams of the respective sample were weighted using the integrated balance of the device, equilibrated at 30 °C for 5 min and then heated to 800 °C with 20 °C/min. The raw weight data were converted to relative weight loss and plotted against the temperature.

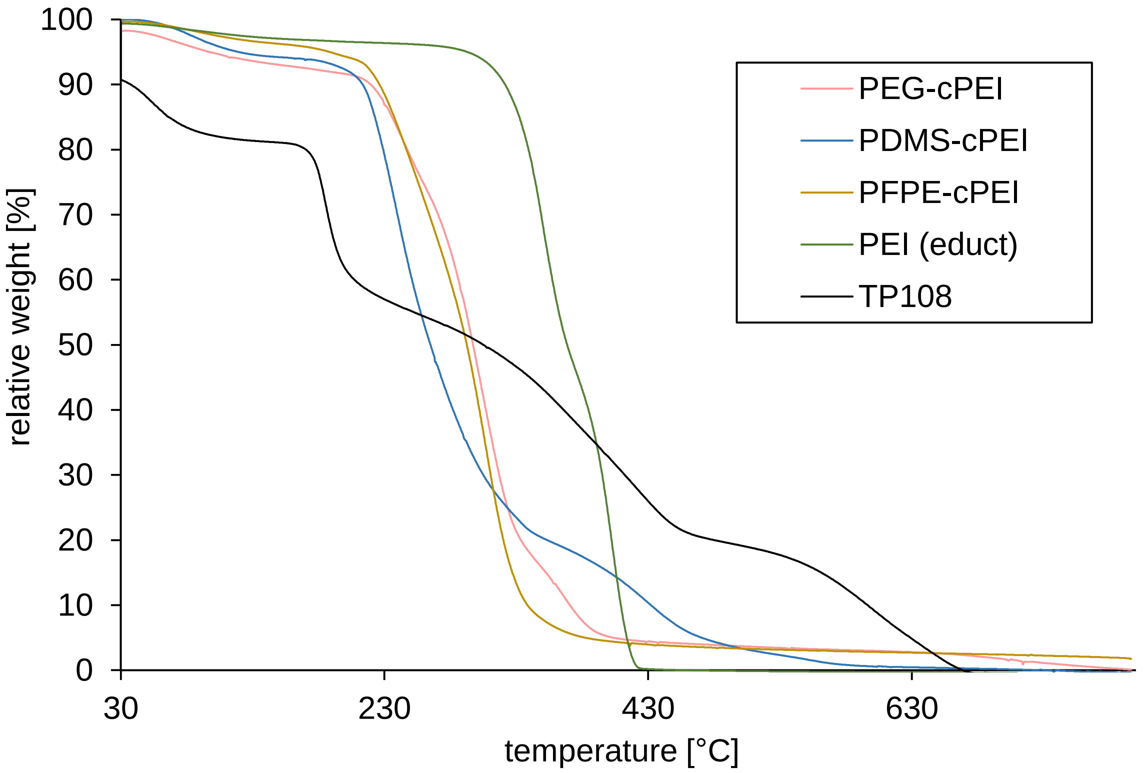

Figure S3. TGA mass loss curves of the cPEI adsorbents, the starting material PEI and TP108 upon heating in nitrogen atmosphere.

Electron Spray Ionization Time of Flight Mass Spectrometry (ESI-ToF-MS)

ESI-ToF-MS was measured on a 6210 ESI-TOF from Agilent (Santa Clara, USA). Solvent flow rate was adjusted to 4 µL/min, spray voltage set to 4 kV. Drying gas flow rate was set to 15 psi (1 bar). All other parameters were adjusted for a maximum abundance of the relative $[M+H]^+$.

Nuclear Magnetic Resonance

NMR spectra were recorded on an ECZ600 spectrometer from JEOL (Freising, Germany) equipped with a 14.09 T magnet at 600.17 MHz (^{1}H), 564.73 MHz (^{19}F) and 150.91 MHz (^{13}C). Spectra were displayed using MestReNova version 14.1.1-24571 from Mestrelab Research

(Santiago de Compostela, Spain). The spectra were manually phase corrected, apodized (exponential: ^{1}H: 0.5 Hz; ^{13}C and ^{19}F: 5.0 Hz), referenced to chloroform (^{1}H: 7.26 ppm; ^{13}C: 77.16 ppm) and the base line was corrected using manually picked points if necessary.

CHN Elemental Analysis

Elemental analysis was carried out on a VARIO EL III instrument from Elementar Analysesysteme (Langenselbold, Germany). The sample was oxidized at 1150 °C and reduced at 850 °C. Carbon dioxide was detected at 100 °C, water at 140 °C and sulfur dioxide at 210 °C.

Table S2. Raw data of the elemental analysis of the cPEI adsorbents. The values of repeated measurements are separated by comma.

sample	C [w%]	N [w%]	H [w%]	S [w%]
TP108	57.36, 44.79, 44.49, 41.68	2.246, 2.141, 2.141, 1.636	11.16, 11.06, 10.53, 8.377	0.357, 0.329, 0.340, 0.252
PEG cross-linker	52.36, 52.27, 52.26	0.006, 0.006, 0.006	8.212, 8.219, 8.389	0.000, 0.000, 0.000
uPEG-cPEI	49.31, 49.04	13.79, 14.54	1.980, 9.441	0.000, 0.000
PEG-cPEI	46.49, 46.29	9.984, 9.981	10.38, 10.15	0.320, 0.288
PDMS cross-linker	40.66, 40.65, 40.64	0.007, 0.126, 0.003	8.596, 8.745, 8.639	0.000, 0.000, 0.000
uPDMS-cPEI	46.84, 47.11	15.15, 15.75	9.805, 9.958	0.000, 0.000
PDMS-cPEI	42.04, 41.96	8.596, 8.916	10.56, 10.24	0.056, 0.035
PFPE cross-linker	32.43, 32.41, 32.92	0.175, 0.150, 0.083	2.841, 2.757, 2.682	0.000, 0.000, 0.000
uPFPE-cPEI	42.00, 41.92	14.49, 14.09	7.019, 6.897	0.000, 0.000
PFPE-cPEI	38.26, 38.23	8.533, 8.558	8.478, 8.476	0.092, 0.078

Fourier Transform Infrared Spectroscopy

Fourier transform infrared spectra were recorded on an Alpha II FT/IR spectrometer from Bruker Optik (Billerica, USA) with platinum attenuated total (ATR) unit (monolithic diamond crystal) and Bruker's OPUS software. 24 scans were recorded with a resolution of 4 cm^{-1}. Spectra were displayed as absorption spectra using MestReNova version 14.1.1-24571. The spectra shown in Figure 1(a) were baseline-corrected using the Whittaker smoother.

Surface Area Determination Using BET Krypton Adsorption

The specific surface area of the adsorbents were calculated in a relative pressure range between $0.13 \leq p/p0 < 0.3$ of the adsorption branch of the krypton adsorption isotherm at 77 K using the multipoint method of Brunauer, Emmet and Teller (BET) [8] with a minimum of five supporting points as described in ISO 9277:2022. [9] The measurement was performed until a relative pressure $p/p0$ of 0.5 using an ASAP 2020 from Micromeritics Instruments (Norcross, USA).

Scanning Electron Microscopy

Scanning electron microscopy of the cPEI adsorbents before sieving was performed on a SU8030 from Hitachi (Berlin, Germany). The samples were adhered to a copper or graphite band and sputtered with gold (5 nm) using a compact coating unit CCU-010 from safematic (Zizers, Switzerland). An acceleration voltage of 15 kV and current of 10 µA was used for the electron beam. The beam, apparatus and X and Y were aligned to minimize the movement of the image. Images of 1280 x 960 size were recorded.

X-ray Photon Spectroscopy

Near ambient pressure X-ray photon spectroscopy (XPS) was performed on indium foil with an EnviroESCA spectrometer from SPECS Surface Nano Analysis (Berlin, Germany) which was equipped with a monochromatic Al Kα X-ray source (excitation energy = 1486.71 eV) and a PHOIBOS 150 electron energy. All spectra were acquired in fixed analyzer transmission (FAT) mode in normal emission with a source-to-sample angle of 60°. The binding energy scale of the instrument was calibrated according to ISO 15472, following a technical procedure provided by SPECS Surface Nano Analysis. The survey spectra were acquired with a pass energy of 100 eV for quantificqation and were quantified utilizing the empirical sensitivity factors that were provided by SPECS Surface Nano Analysis (the sensitivity factors were corrected with the transmission function of the spectrometer). All binding energies were

calibrated to the signal observed for the aliphatic C–C bond component (E_{bind} = 285 eV) if not stated otherwise.

Table S3. Raw data of the synthesis and elemental compositions obtained by XP survey spectra quantification (incl. their standard deviation) of the charged adsorbents.

sample	C [at%]	N [at%]	O [at%]	F [at%]	Si [at%]	Cl [at%]	In [at%]
TP108	89.86 ± 0.77	2.69 ± 0.66	4.37 ± 0.43			3.08 ± 0.20	
PEG-cPEI	67.55 ± 0.51	10.63 ± 0.42	15.11 ± 0.36			6.71 ± 0.19	
PDMS-cPEI	61.88 ± 0.49	8.95 ± 0.40	12.23 ± 0.29		9.15 ± 0.30	7.80 ± 0.18	
PFPE-cPEI	54.80 ± 0.45	9.28 ± 0.35	10.57 ± 0.31	17.08 ± 0.27		7.77 ± 0.16	0.51 ± 0.04

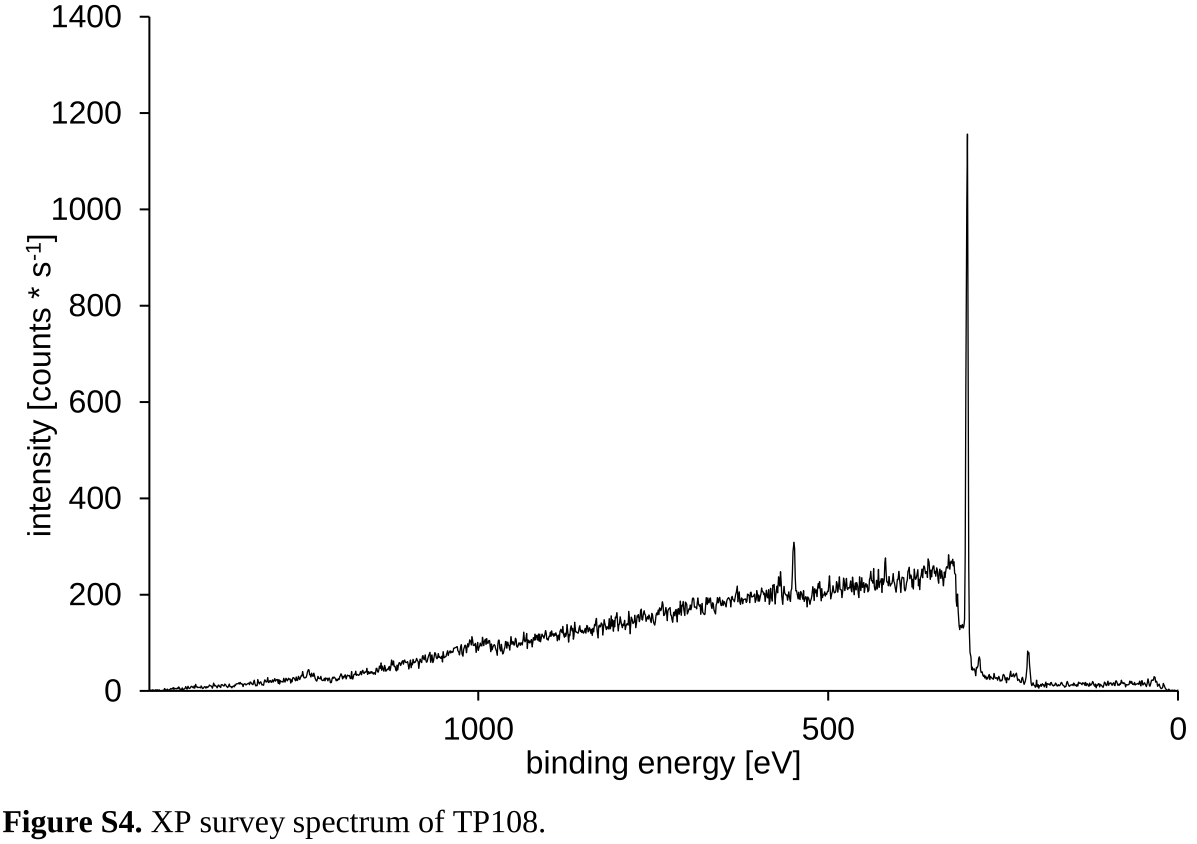

Figure S4. XP survey spectrum of TP108.

Infrared Spectra

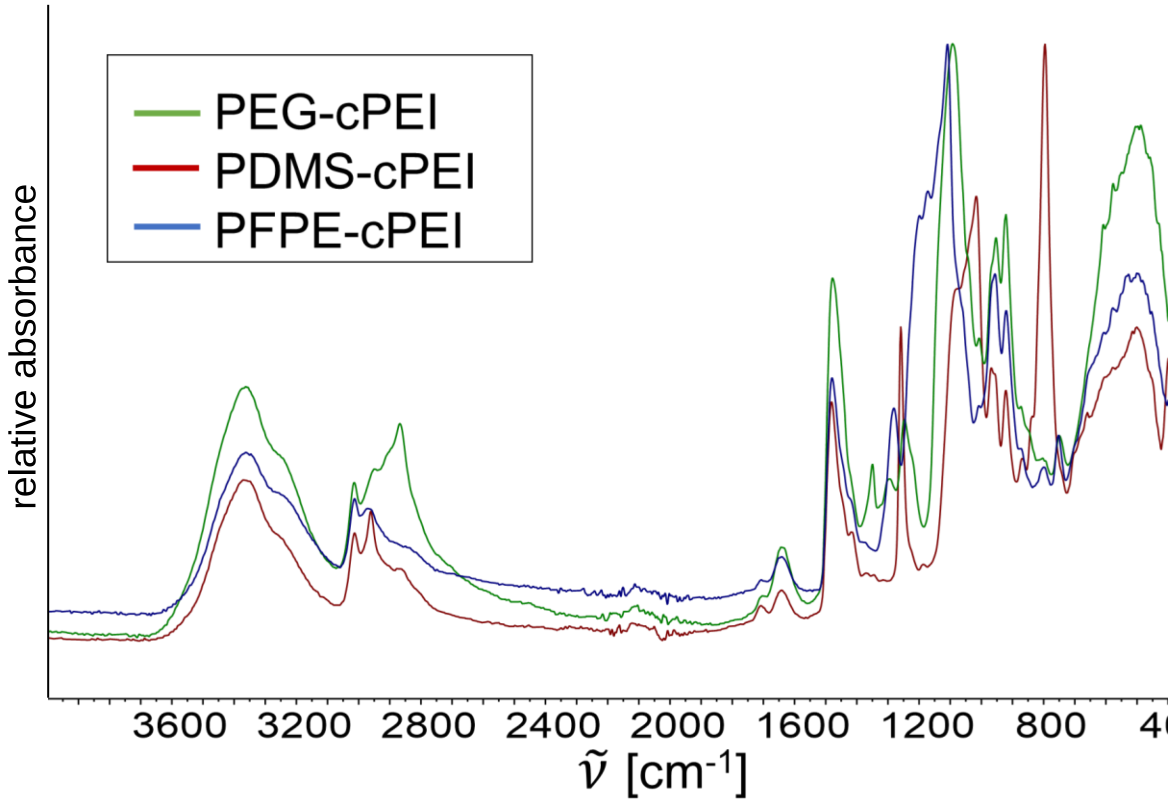

Figure S5. IR spectra of the cPEI adsorbents.

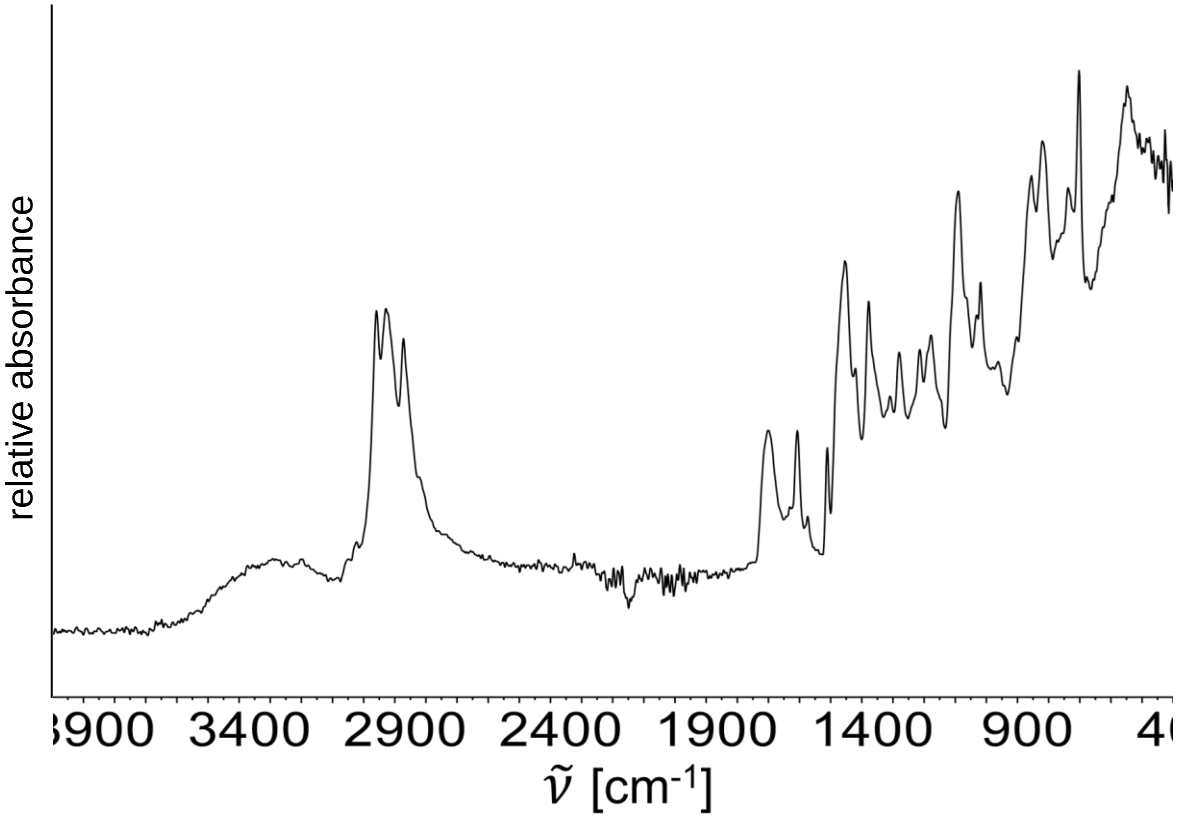

Figure S6. IR spectrum of TP108.

NMR Spectra

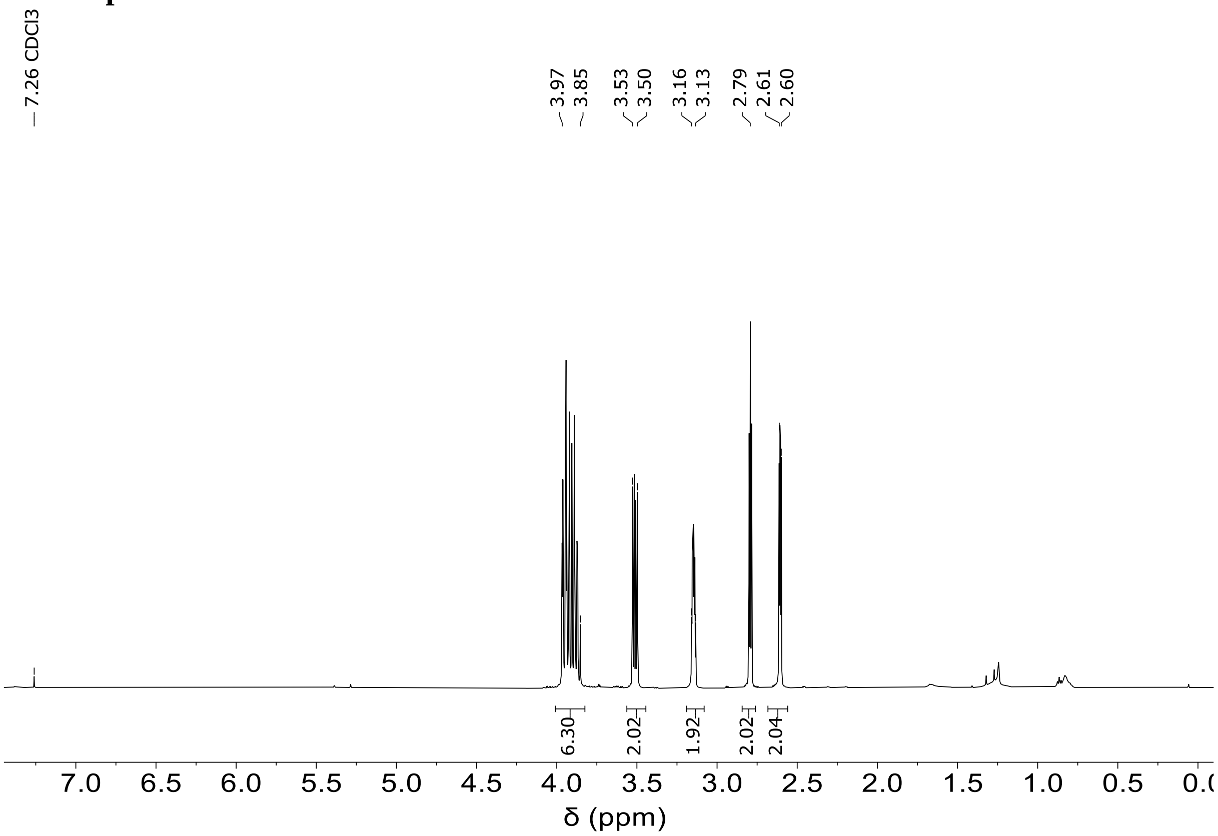

Figure S7. ^{1}H NMR spectrum of the PFPE cross-linker.

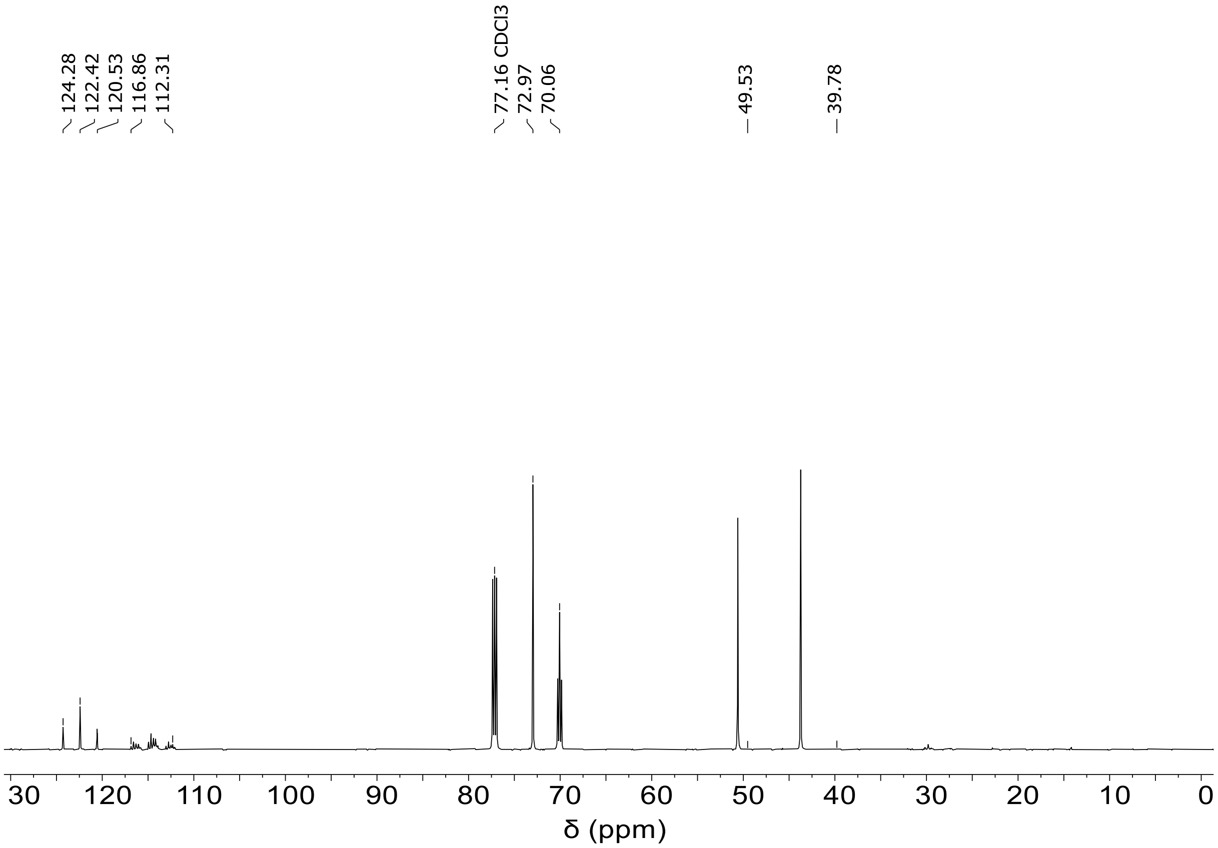

Figure S8. ^{13}C NMR spectrum of the PFPE cross-linker.

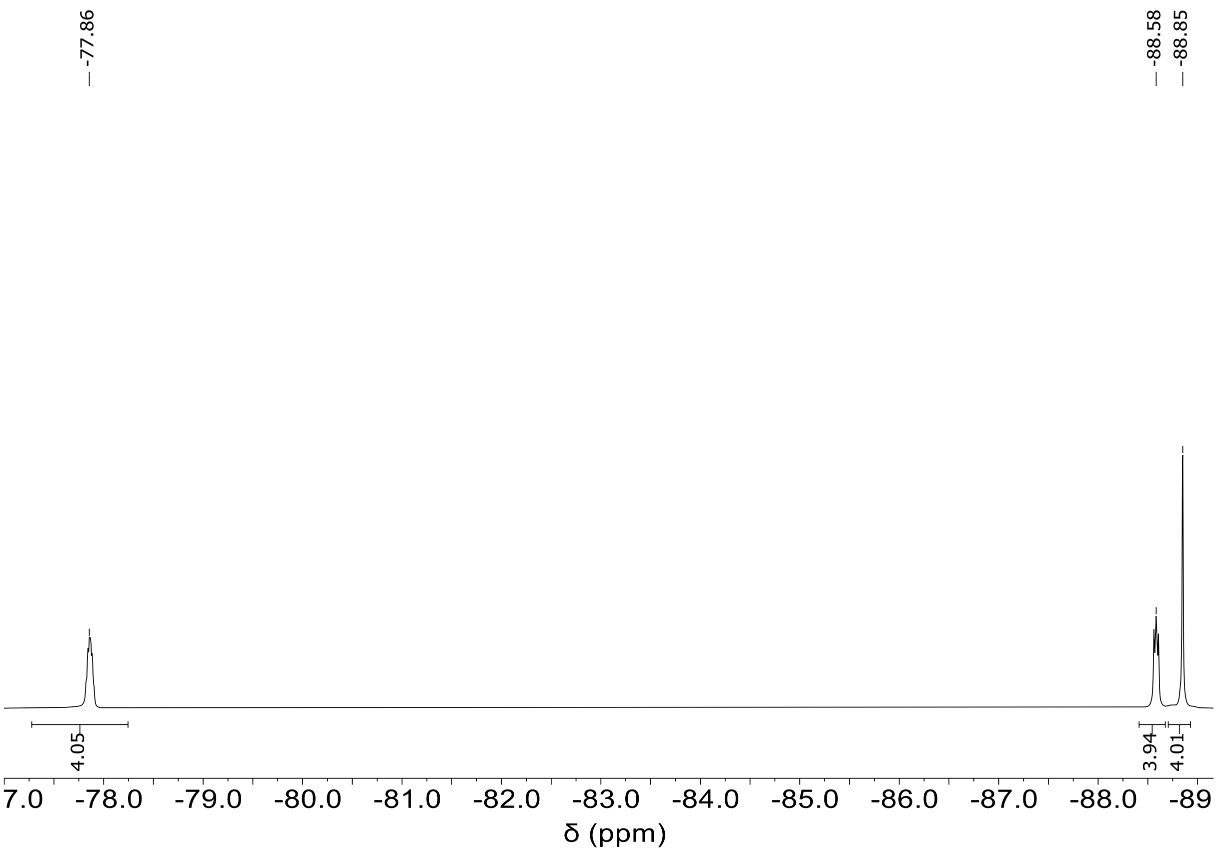

Figure S9. ^{19}F NMR spectrum of the PFPE cross-linker.

Mass Spectra

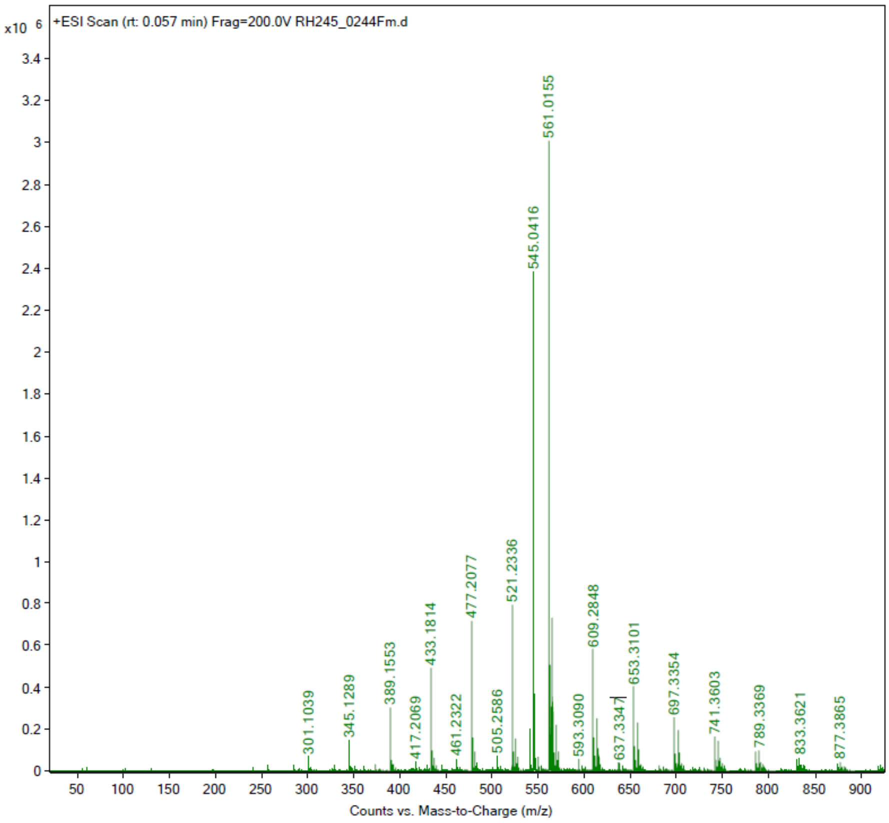

Figure S10. ESI-ToF-MS of the PFPE cross-linker.

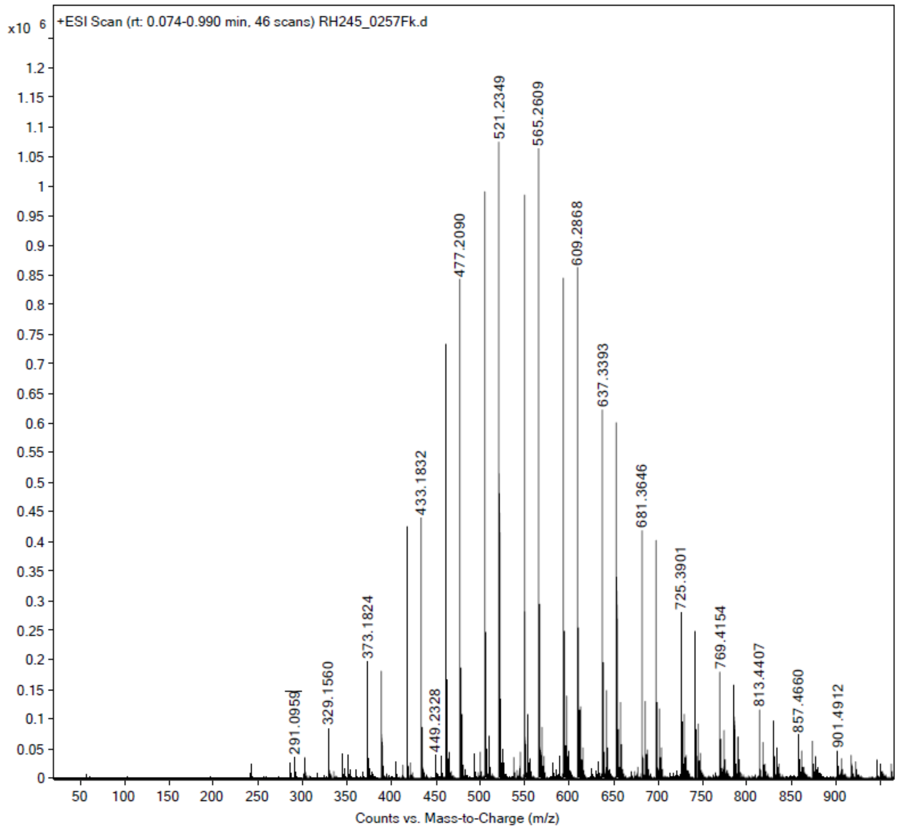

Figure S11. ESI-ToF-MS of the PEG cross-linker.

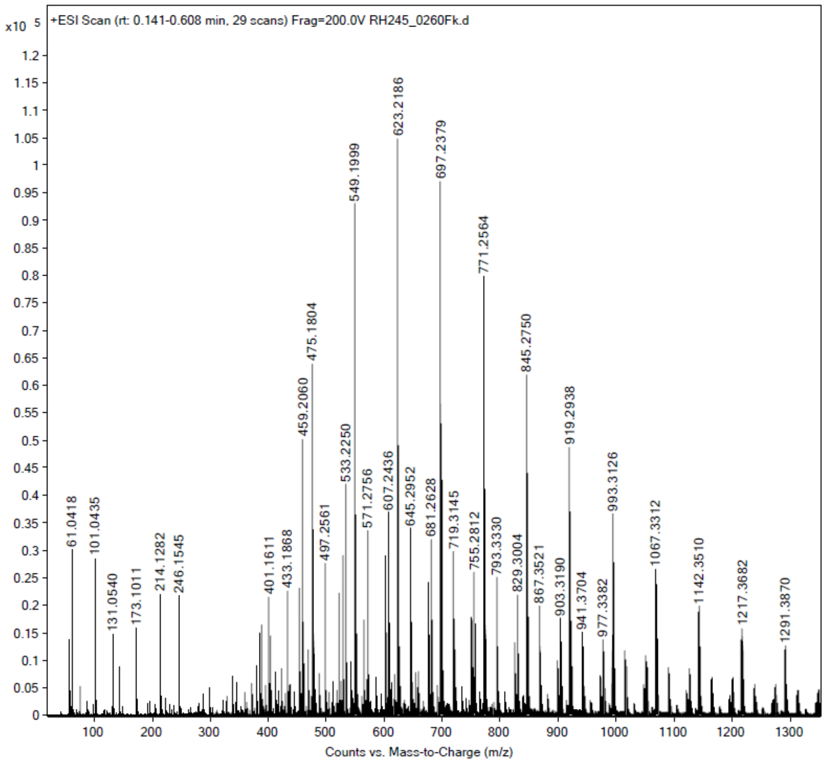

Figure S12. ESI-ToF-MS of the PDMS cross-linker.

BET Isotherms

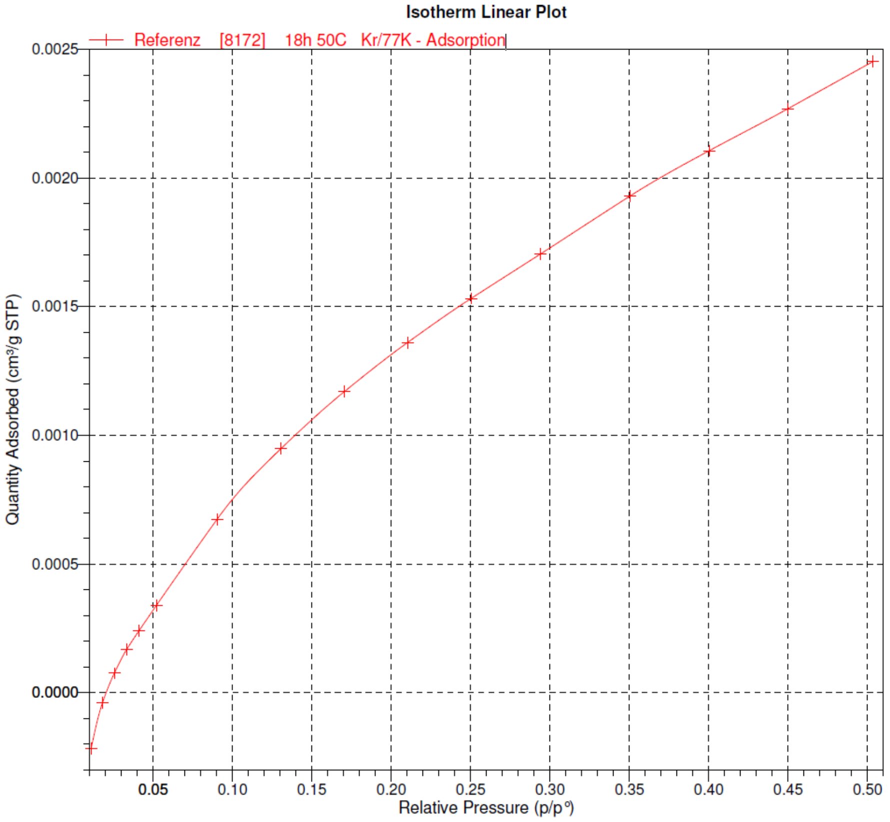

Figure S14. Krypton adsorption isotherm of TP108. This resulting surface area (0.0088 ± 0.0003) m^2/g is close to the detection limit.

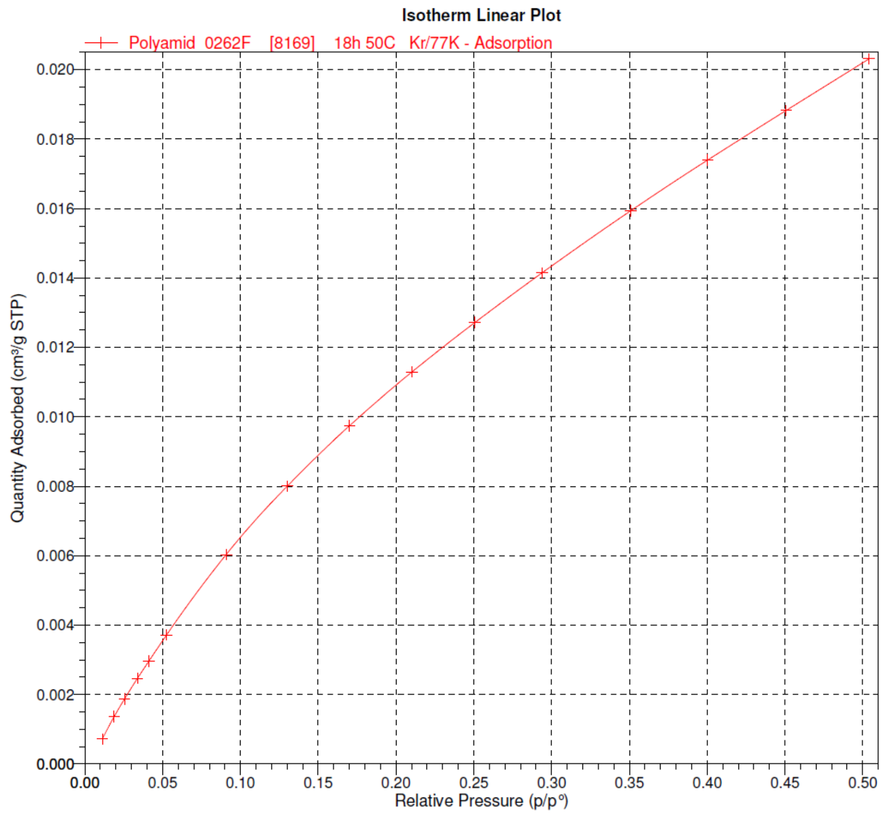

Figure S15. Krypton adsorption isotherm of PEG-cPEI.

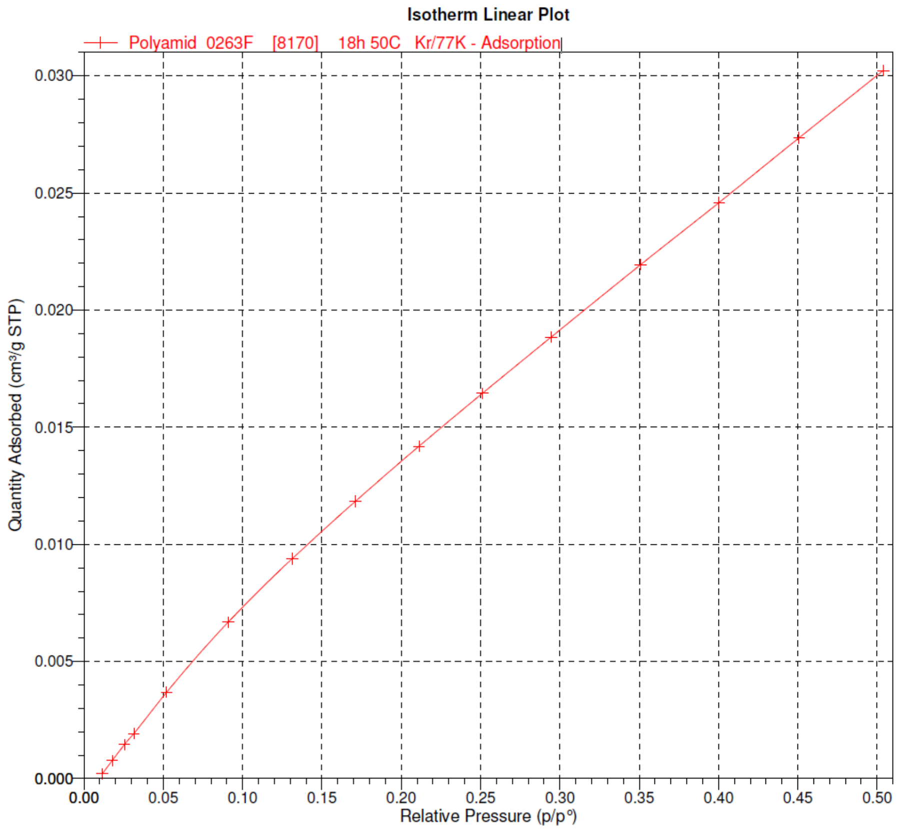

Figure S16. Krypton adsorption isotherm of PDMS-cPEI.

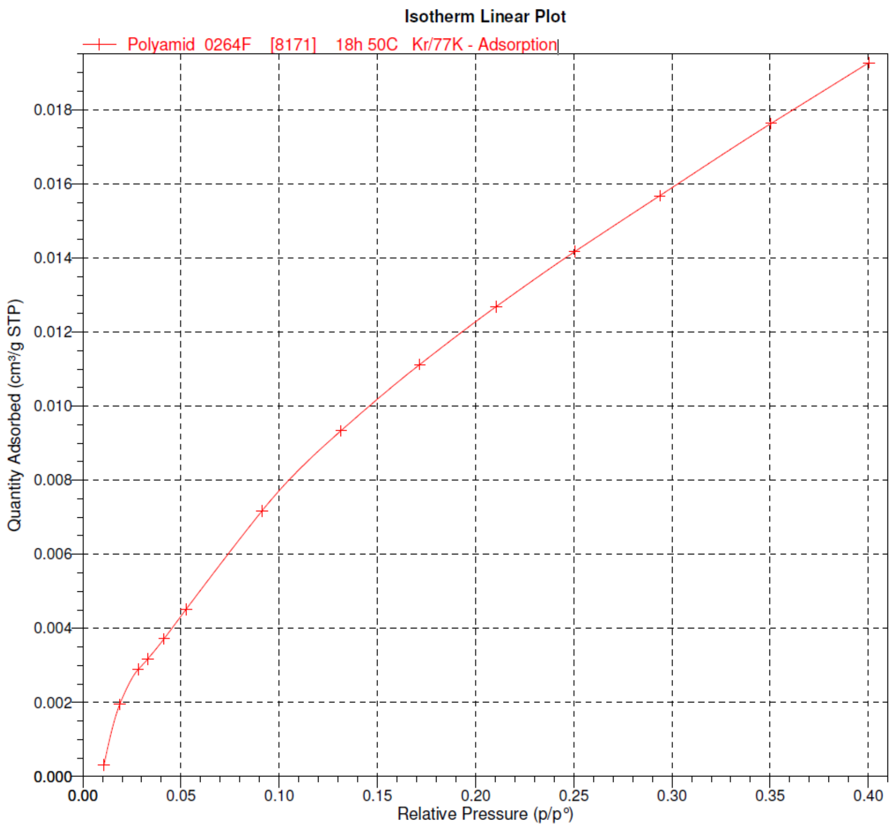

Figure S17. Krypton adsorption isotherm of PFPE-cPEI.

Table S4. Further details of the BET measurement.

Adsorbent	Slope [g cm^{-3}]	Intercept [g cm^{-3}]	Qm [g cm^{-3}]
TP108	527 ± 19	87 ± 4	0.0016
PEG-cPEI	66 ± 2	10.0 ± 0.3	0.0132
PDMS-cPEI	37.0 ± 0.8	11.1 ± 0.2	0.0208
PFPE-cPEI	63 ± 1	7.8 ± 0.2	0.0140

Light Microscopic Images (selection)

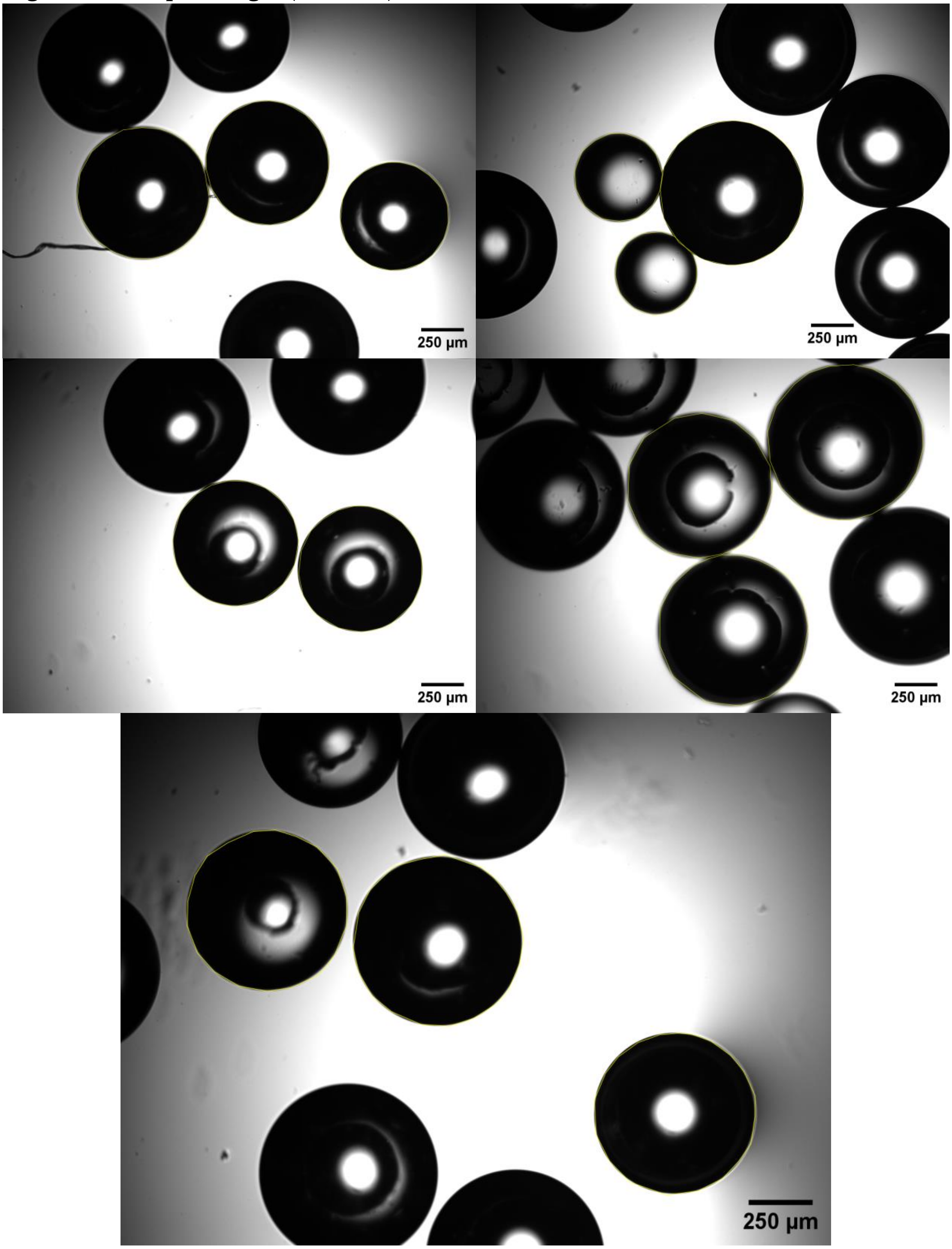

Figure S18. Light microscopic images of TP108. The yellow lines indicate the particle boundaries that were set for the analysis of the particle size distribution.

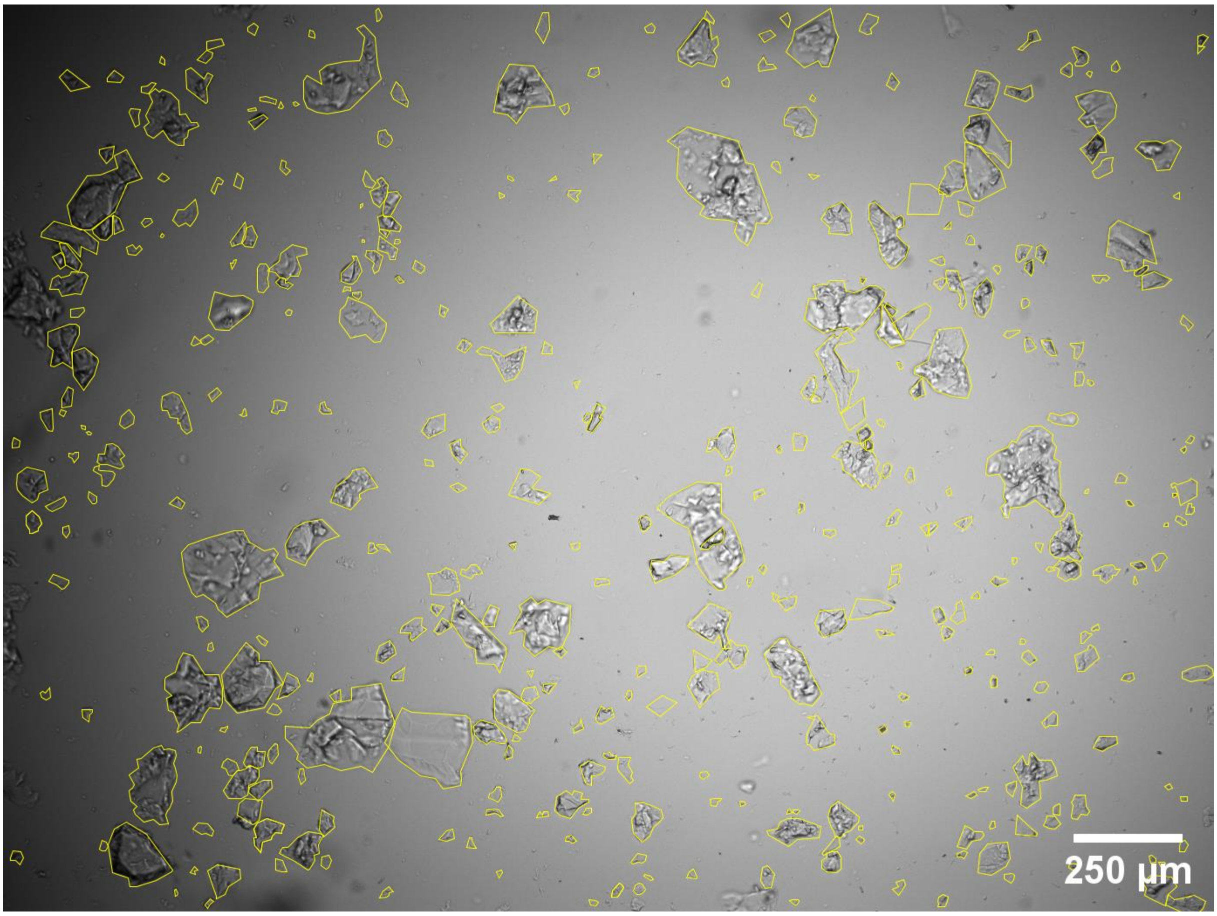

Figure S19. Light microscopic image of PEG-cPEI. The yellow lines indicate the particle boundaries that were set for the analysis of the particle size distribution.

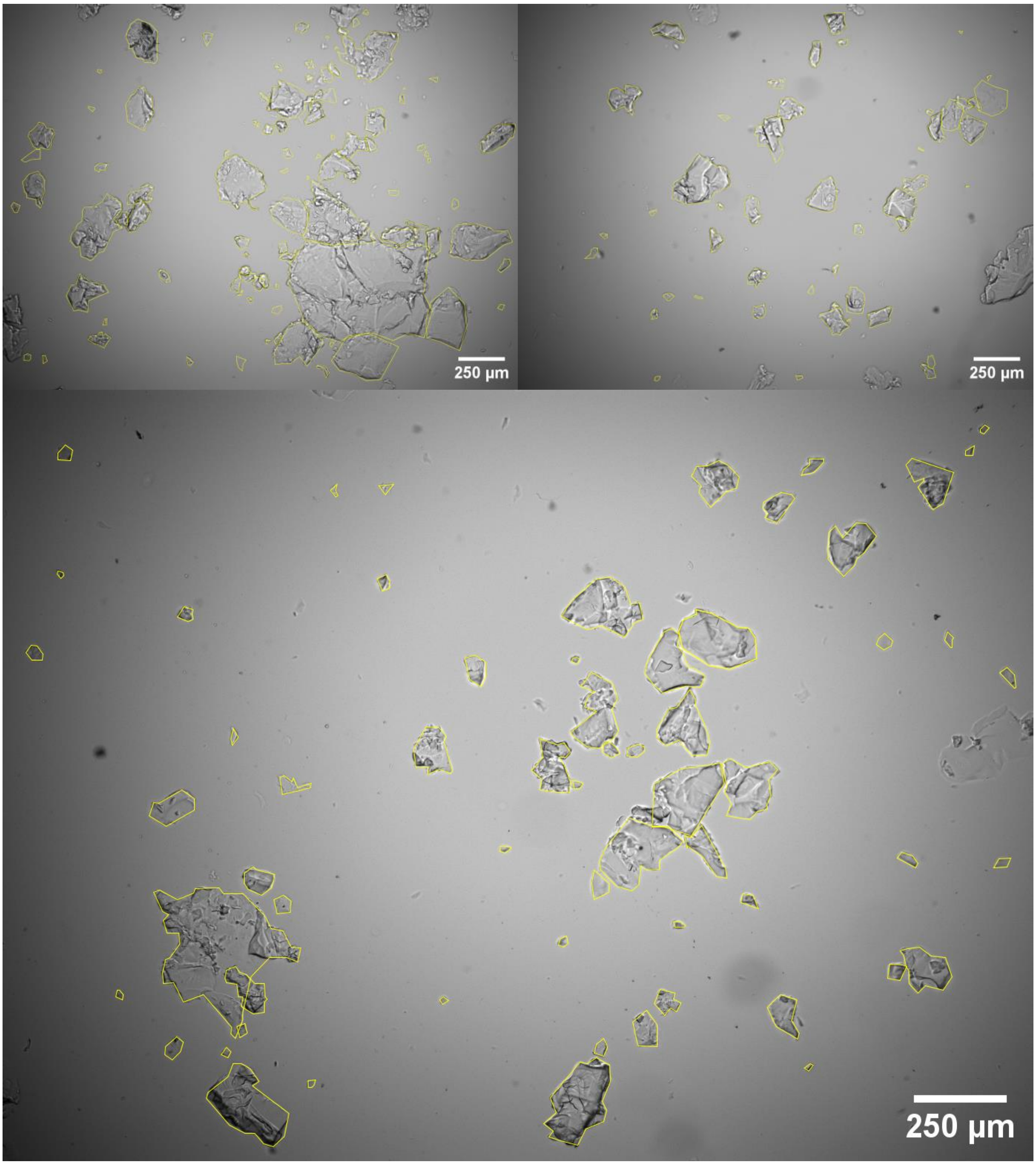

Figure S20. Light microscopic images of PDMS-cPEI. The yellow lines indicate the particle boundaries that were set for the analysis of the particle size distribution.

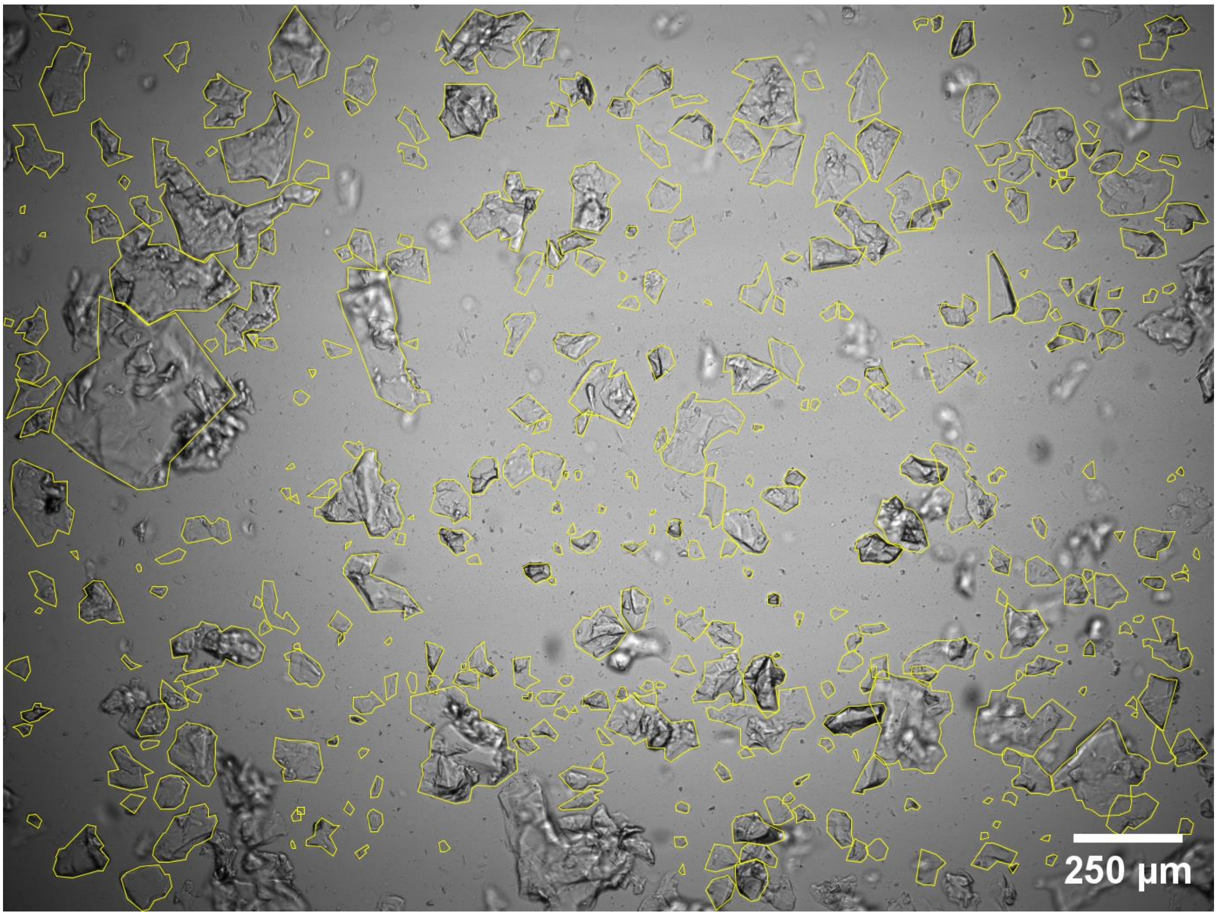

Figure S21. Light microscopic image of PFPE-cPEI. The yellow lines indicate the particle boundaries that were set for the analysis of the particle size distribution.

Histograms of the Particle Size Distribution

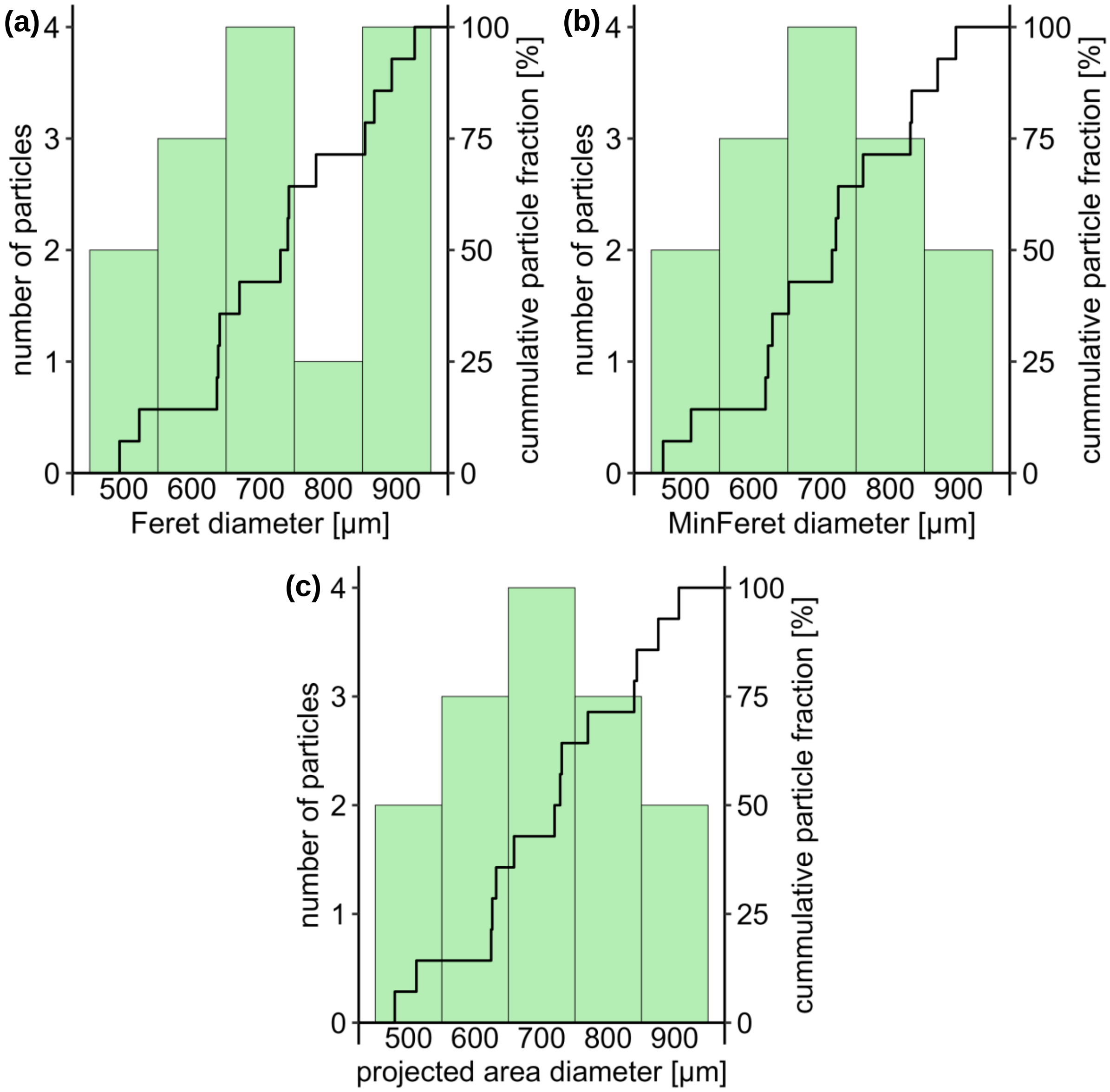

Figure S22. Number-weighted histograms including cumulative curves of the size distribution of **(a)** the Feret diameter,**(b)** the minFeret diameter and **(c)** the projected area diameters of TP108. The histograms were obtained by analysis of the particle areas (n = 14) in light microscopic images.

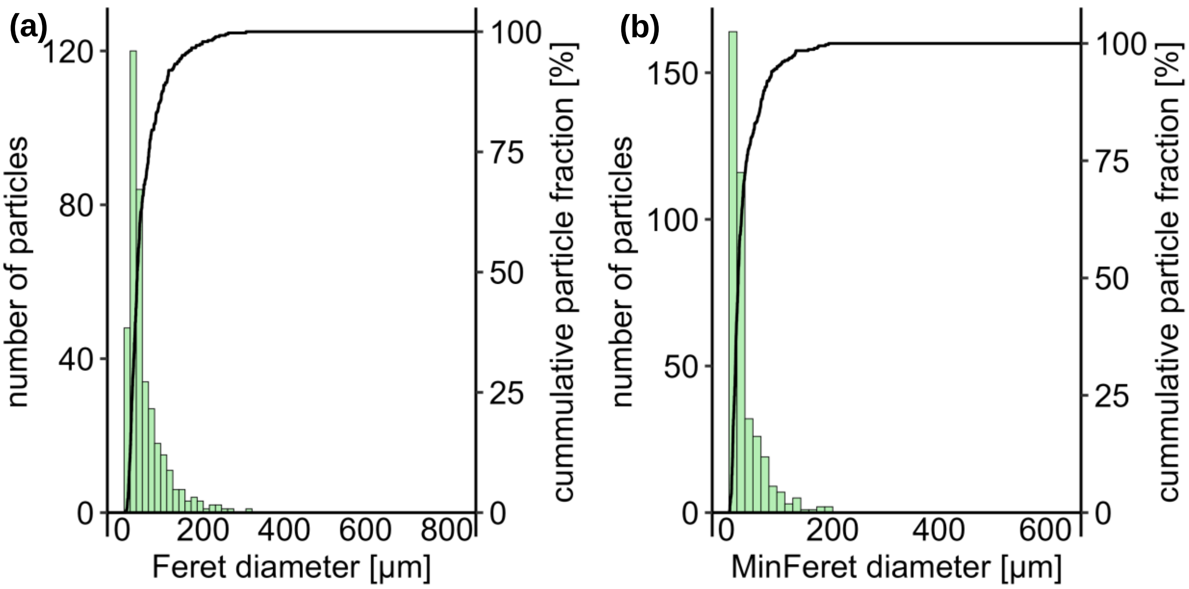

Figure S23. Number-weighted histograms including cumulative curves of the size distribution of **(a)** the Feret diameter and **(b)** the minFeret diameter of PEG-cPEI. The histograms were obtained by analysis of the particle areas (n = 387) in light microscopic images.

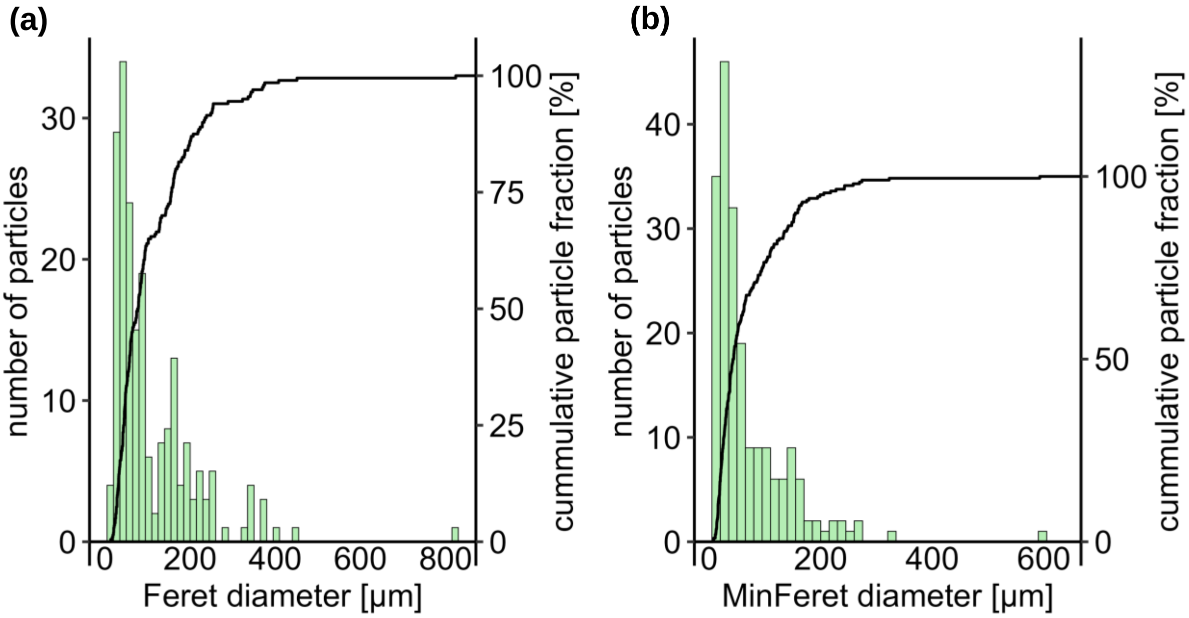

Figure S24. Number-weighted histograms including cumulative curves of the size distribution of **(a)** the Feret diameter and **(b)** the minFeret diameter of PDMS-cPEI. The histograms were obtained by analysis of the particle areas (n = 200) in light microscopic images.

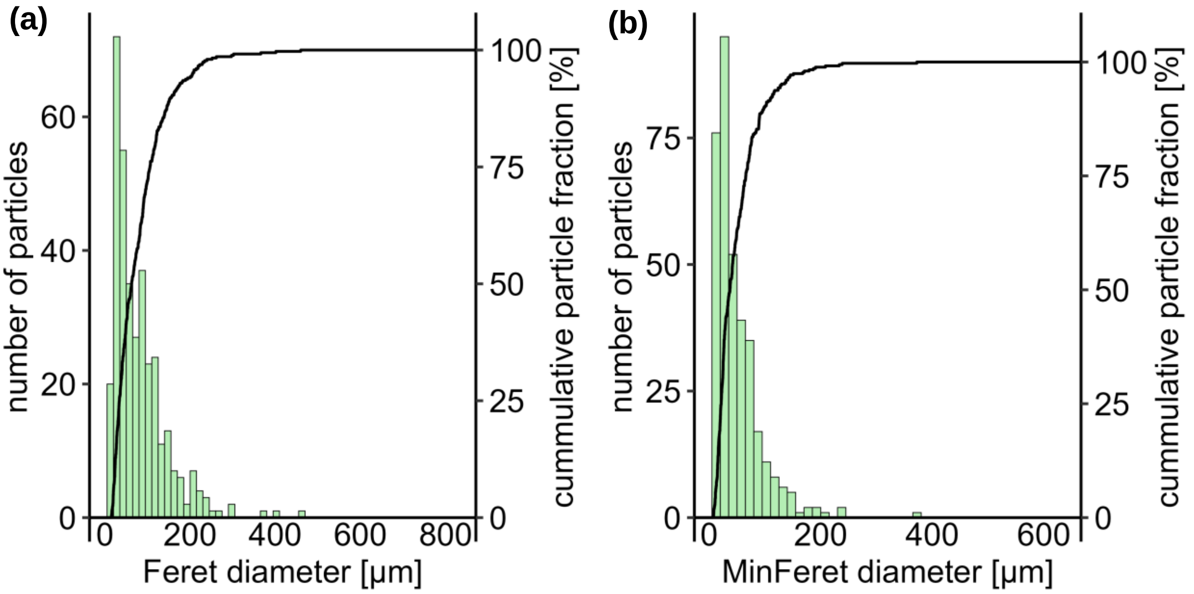

Figure S25. Number-weighted histograms including cumulative curves of the size distribution of **(a)** the Feret diameter and **(b)** the minFeret diameter of PFPE-cPEI. The histograms were obtained by analysis of the particle areas (n = 353) in light microscopic images.

Scanning Electron Microscopic (SEM) Images

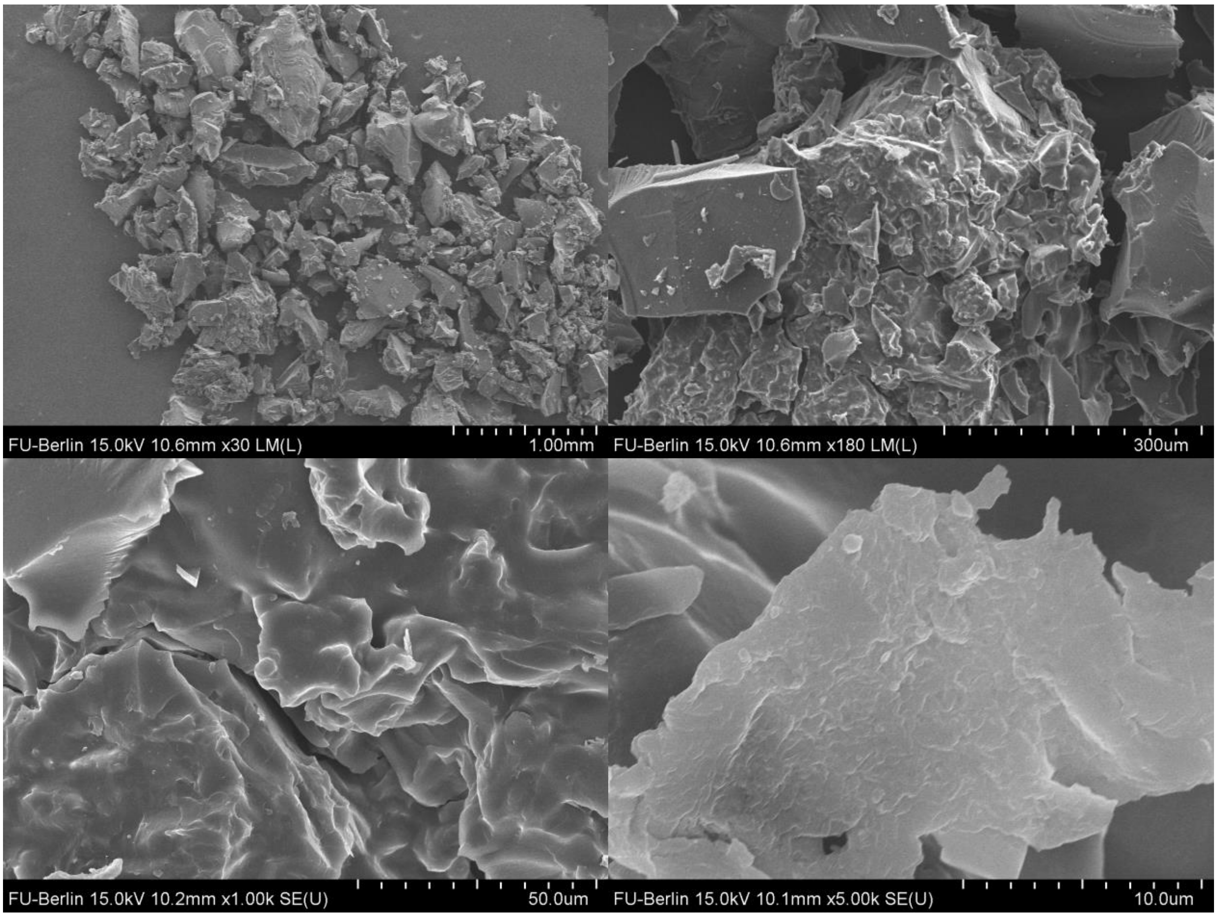

Figure S26. SEM images of uPEG-cPEI.

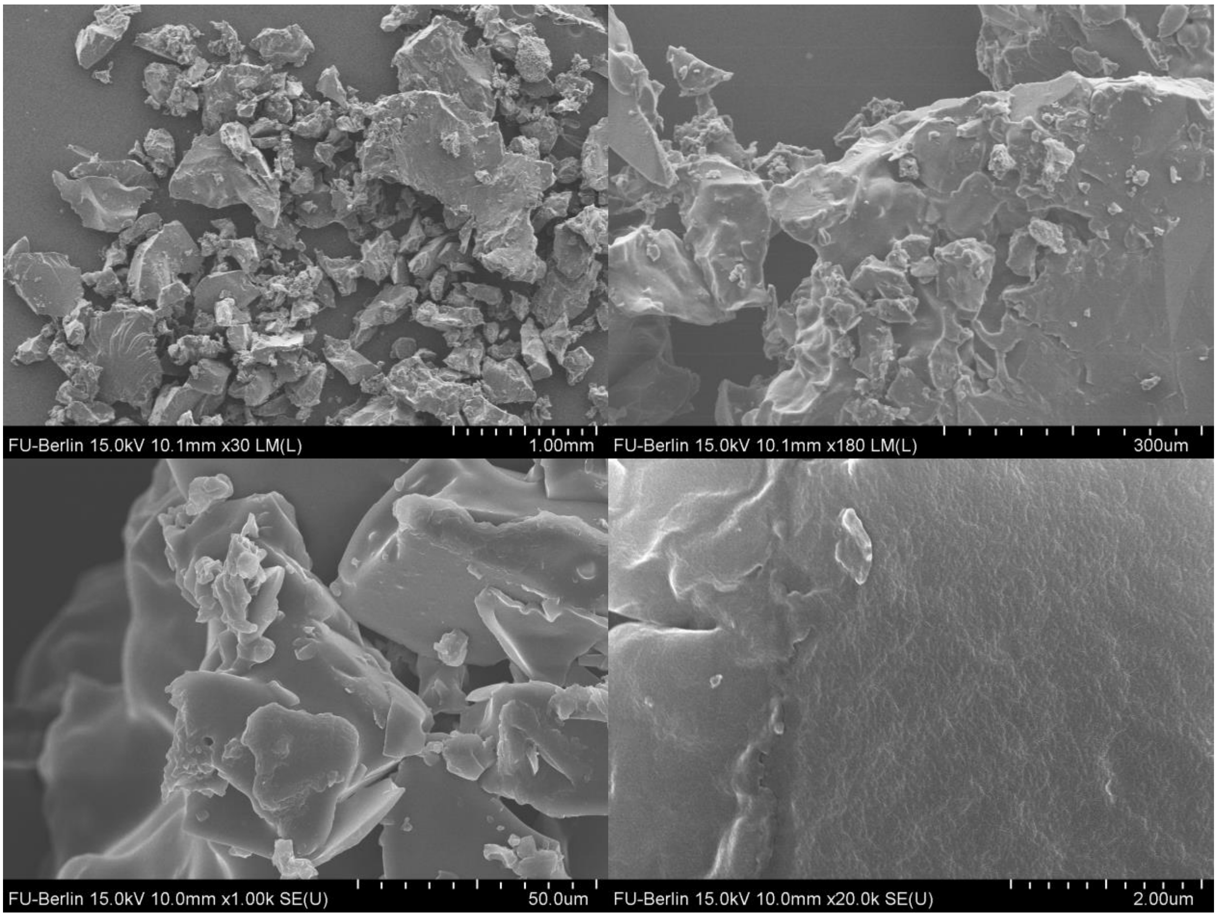

Figure S27. SEM images of uPDMS-cPEI.

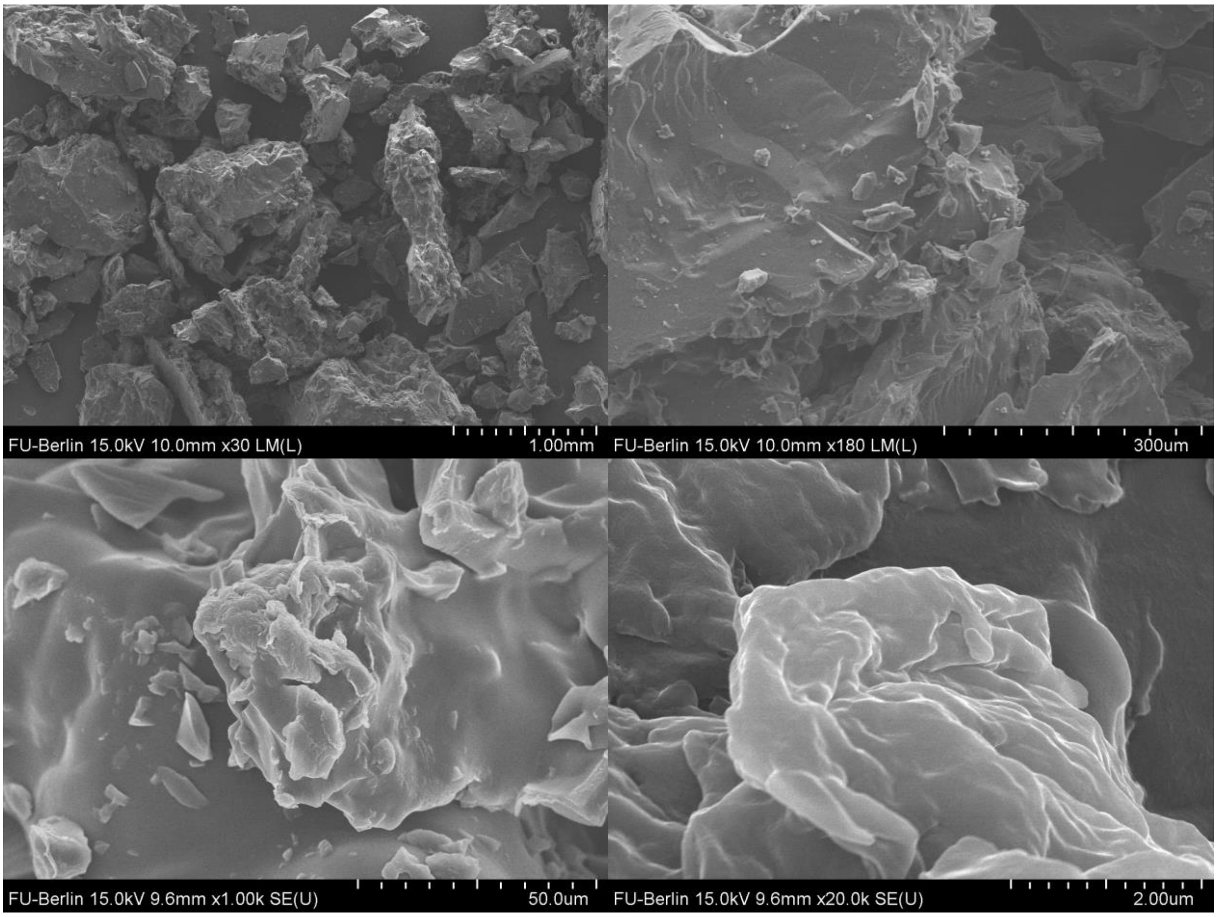

Figure S28. SEM images of uPFPE-cPEI.

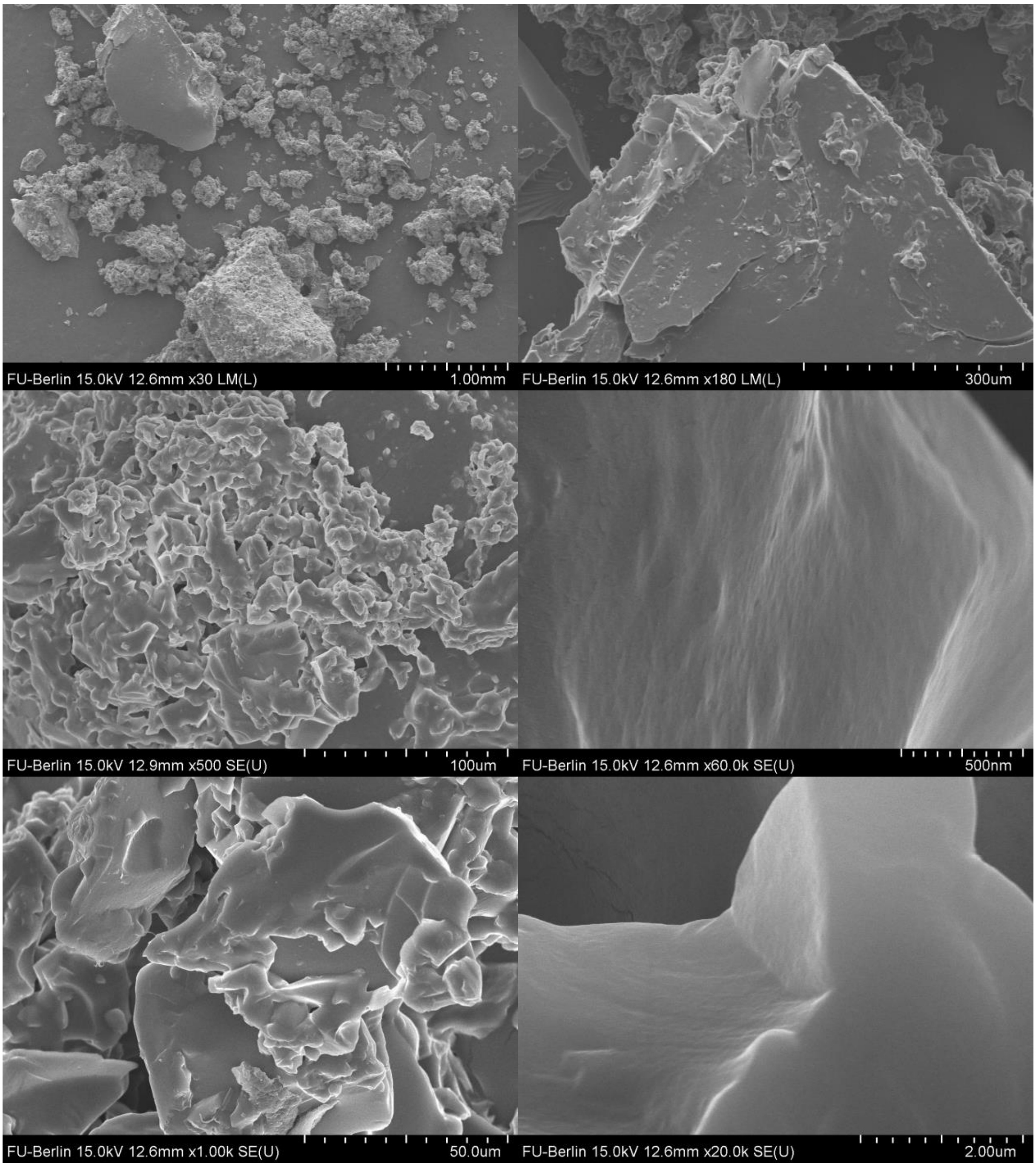

Figure S29. SEM images of PEG-cPEI.

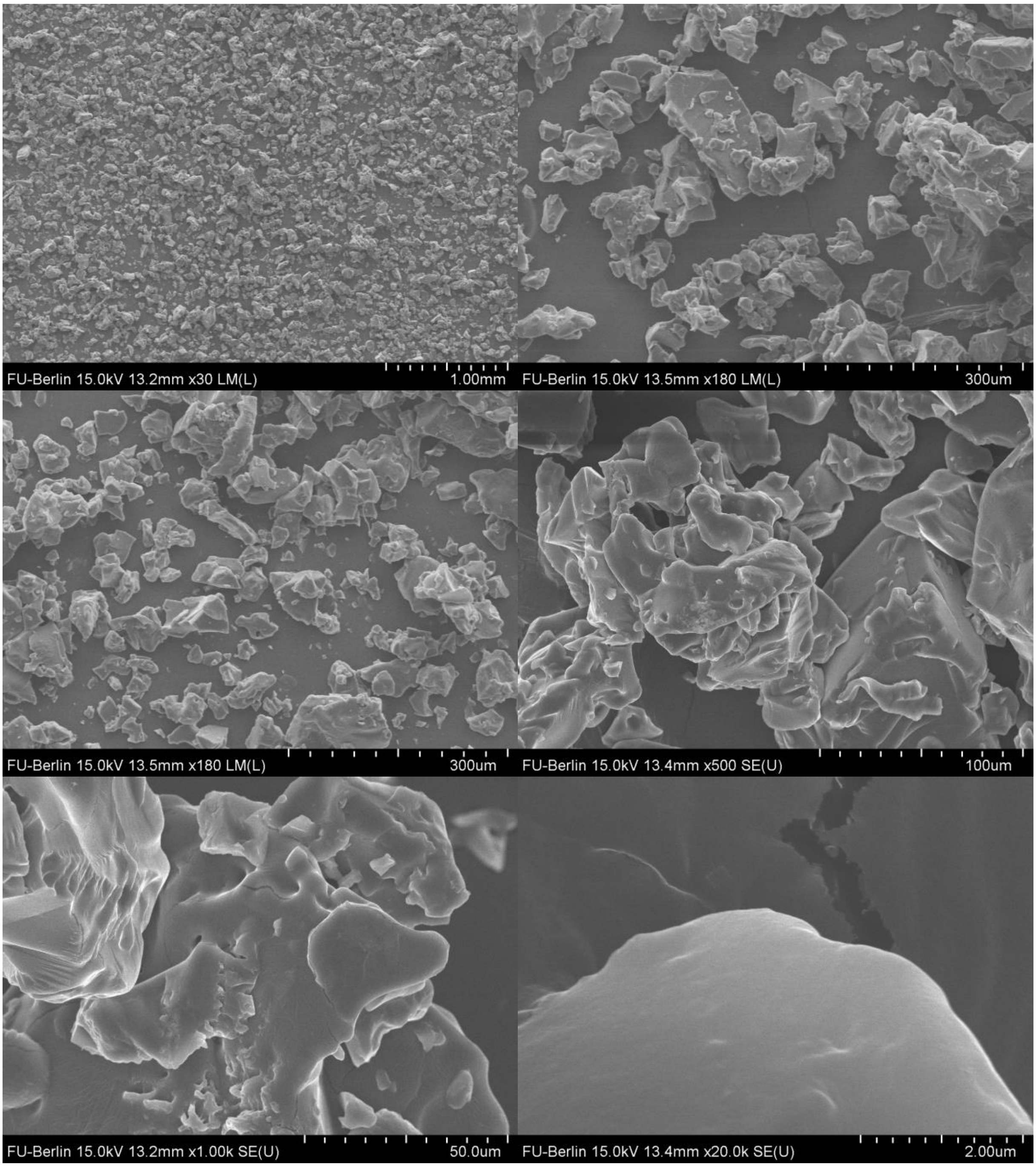

Figure S30. SEM images of PDMS-cPEI.

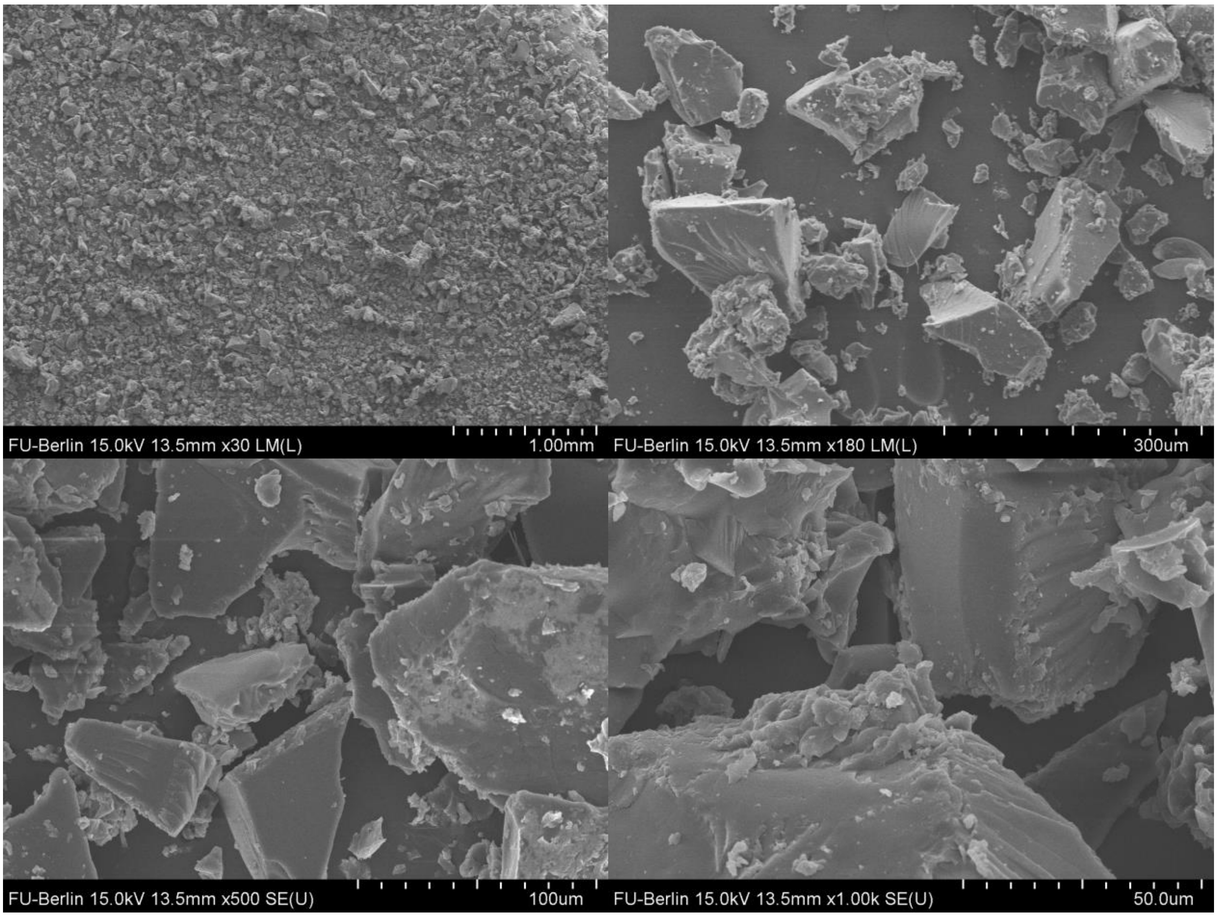

Figure S31. SEM images of PFPE-cPEI.

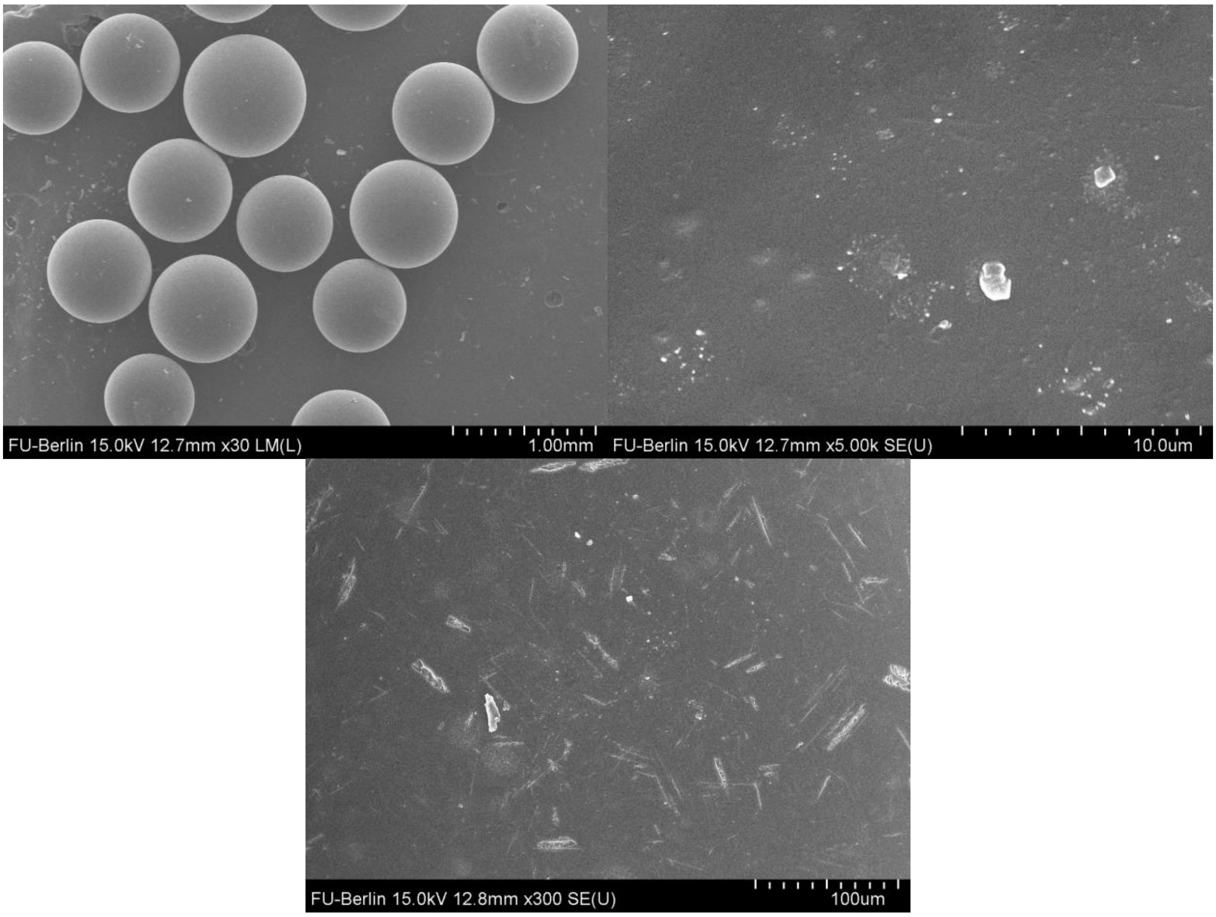

Figure S32. SEM images of TP108.

Single Point Adsorption Coefficients

Table S5. Single point adsorption coefficients at 80 mg/L adsorbent dose in L/kg.

	adsorbent		
**PFAS**	**TP108**	**PFPE-cPEI**	**PEG-cPEI**
PFBA	0.8588	0.0157	0.0012
PFHxA	0.9551	0.1701	0.0051
TFMSA	3.6968	0.0190	0.0032
PFPeS	0.3140	2.5525	0.0174

References

(1) *1.11106.0001 MQuant Chloride Test*. Merck KGaA, https://www.merckmillipore.com/DE/de/product/Chloride-Test,MDA_CHEM-111106#documentation (accessed 2025-01-16).

(2) Lafarge, T.; Possolo, A. *Uncertainty Machine --- User's Manual*. National Institute of Standards and Technology, 2013. https://tsapps.nist.gov/publication/get_pdf.cfm?pub_id=913874 (accessed 2025-01-16).

(3) Lafarge, T.; Possolo, A. The NIST Uncertainty Machine. *NCSLI Measure* **2016**, *10* (3), 20-27. DOI: 10.1080/19315775.2015.11721732.

(4) Smales, G. J.; Pauw, B. R. The MOUSE project: a meticulous approach for obtaining traceable, wide-range X-ray scattering information. *J. Instrum.* **2021**, *16* (06), P06034. DOI: 10.1088/1748-0221/16/06/p06034.

(5) Pauw, B. R.; Smith, A. J.; Snow, T.; Terrill, N. J.; Thunemann, A. F. The modular small-angle X-ray scattering data correction sequence. *J. Appl. Crystallogr.* **2017**, *50*, 1800-1811. DOI: 10.1107/S1600576717015096.

(6) Filik, J.; Ashton, A. W.; Chang, P. C. Y.; Chater, P. A.; Day, S. J.; Drakopoulos, M.; Gerring, M. W.; Hart, M. L.; Magdysyuk, O. V.; Michalik, S.; et al. Processing two-dimensional X-ray diffraction and small-angle scattering data in DAWN 2. *J. Appl. Crystallogr.* **2017**, *50*, 959-966. DOI: 10.1107/S1600576717004708.

(7) Bressler, I.; Pauw, B. R.; Thunemann, A. F. McSAS: software for the retrieval of model parameter distributions from scattering patterns. *J. Appl. Crystallogr.* **2015**, *48*, 962-969. DOI: 10.1107/S1600576715007347.

(8) Brunauer, S.; Emmett, P. H.; Teller, E. Adsorption of Gases in Multimolecular Layers. *J. Am. Chem. Soc.* **2002**, *60* (2), 309-319. DOI: 10.1021/ja01269a023.

(9) International Organization for Standardization, *Determination of the specific surface area of solids by gas adsorption - BET method, ISO 9277:2022*; Geneva, 2022.
